# Expansion and Refinement of Deep Sequence-Coupled Biopanning Technology for Epitope-Specific Antibody Responses in Human Serum

**DOI:** 10.3390/v12101114

**Published:** 2020-09-30

**Authors:** Nikole L. Warner, Alexandria C. Linville, Susan B. Core, Brechla Moreno, Juan M. Pascale, David S. Peabody, Bryce Chackerian, Kathryn M. Frietze

**Affiliations:** 1 Department and Molecular Genetics and Microbiology, University of New Mexico Health Sciences, Albuquerque, NM 87131, USA; nlwarner@salud.unm.edu (N.L.W.); alexandria.linville@huskers.unl.edu (A.C.L.); score@salud.unm.edu (S.B.C.); dpeabody@salud.unm.edu (D.S.P.); bchackerian@salud.unm.edu (B.C.); 2 Gorgas Memorial Institute, Panama 0801, Panama; bmoreno@gorgas.gob.pa (B.M.); jmpascale@gorgas.gob.pa (J.M.P.); 3 Clinical and Translational Science Center, University of New Mexico Health Sciences, Albuquerque, NM 87131, USA

**Keywords:** antibody, dengue virus, biopanning, MS2, bacteriophage, affinity selection, virus-like particle

## Abstract

Identifying the specific epitopes targeted by antibodies elicited in response to infectious diseases is important for developing vaccines and diagnostics. However, techniques for broadly exploring the specificity of antibodies in a rapid manner are lacking, limiting our ability to quickly respond to emerging viruses. We previously reported a technology that couples deep sequencing technology with a bacteriophage MS2 virus-like particle (VLP) peptide display platform for identifying pathogen-specific antibody responses. Here, we describe refinements that expand the number of patient samples that can be processed at one time, increasing the utility of this technology for rapidly responding to emerging infectious diseases. We used dengue virus (DENV) as a model system since much is already known about the antibody response. Sera from primary DENV-infected patients (n = 28) were used to pan an MS2 bacteriophage VLP library displaying all possible 10-amino-acid peptides from the DENV polypeptide. Selected VLPs were identified by deep sequencing and further investigated by enzyme-linked immunosorbent assay. We identified previously described immunodominant regions of envelope and nonstructural protein-1, as well as a number of other epitopes. Our refinement of the deep sequence-coupled biopanning technology expands the utility of this approach for rapidly investigating the specificity of antibody responses to infectious diseases.

## 1. Introduction

Identifying the epitope specificity of antibodies elicited in response to infection can be a valuable component in vaccine development. Methods such as phage display and peptide scanning have been useful for identifying the epitopes of monoclonal antibodies and targets of antibodies in polyclonal sera, but these methods are not suitable for assessing many samples and can be time consuming or expensive to use. We have developed a technology, called deep sequence-coupled biopanning (DSCB), to interrogate the specificity of human polyclonal serum antibody responses. We have used DSCB to map epitopes targeted by antibodies in ovarian cancer patients, dengue virus infected individuals, and women with *Chlamydia trachomatis* infection [1,2,3]. This technology is based on a novel affinity selection platform in which peptides are displayed on the surface of bacteriophage MS2 virus-like particles (VLPs) [4]. Briefly, random peptides or overlapping peptides derived from antigens of interest are displayed in a constrained beta-hairpin loop such that 90 total copies of a peptide are displayed per VLP. These VLPs also encapsidate their own coding mRNA, meaning that upon affinity selection using antibodies, selected VLPs can be readily identified by RT-PCR and sequencing. Coupling this technology with deep sequencing allows for detailed and in-depth identification of specific epitopes targeted by antibodies in human sera. However, one limitation of the prototype version of this technology has been the requirement to clone recovered affinity-selected nucleic acid sequences into a plasmid prior to deep sequencing. Here, we describe our efforts to streamline the DSCB technology so that it is more efficient, allowing multiple samples to be processed at once. As a model, we used serum samples from dengue-virus-infected individuals because the antibody response to dengue virus (DENV) has been extensively characterized.

DENV, a flavivirus transmitted by *Aedes* mosquitoes, infects approximately 400 million people, in over 125 countries and causes 10,000 deaths annually [5,6,7]. The antibody response to DENV has been extensively characterized and includes antibodies to both structural and non-structural proteins. DENV is comprised of four serotypes, DENV1–4. After first exposure to a specific serotype of DENV, an individual develops long-lasting protective antibodies to the homologous serotype [8,9,10]. However, upon subsequent infection with a second serotype of DENV (secondary infection) individuals have a higher risk of developing severe dengue (SD); dengue hemorrhagic fever (DHF), or dengue shock syndrome (DSS) [8,9,10]. This increased risk of severe disease is thought to be caused in part by antibody-dependent enhancement (ADE) of infection, whereby non-neutralizing antibodies largely targeting conserved epitopes within the pre-membrane (prM) and envelope (E), bind to DENV virions, allowing the virus to infect cells that express Fcγ receptors [8,10]. Additionally, antibodies targeting non-structural protein 1 (NS1), a viral protein secreted by DENV-infected cells, have also been implicated in the pathogenesis of DENV [11,12,13,14]. Of particular interest is the NS1 wing domain, which has been characterized as an immunodominant region of the protein [15,16]. Antibodies directed against the wing domain cross react with host proteins, leading to an autoantibody-like response that may contribute to pathogenesis [17,18,19,20,21,22,23,24,25,26].

Here, we used serum from individuals acutely infected with primary DENV and investigated the antibody response using DSCB technology. A DENV-specific library of peptides displayed on bacteriophage MS2 VLPs was panned against IgG isolated from patient samples. Our studies include refinements of the DSCB method that allow us to process up to sixty samples at one time, in the same time frame (one week) as using our previous method. This modification expands the utility of DSCB such that it can rapidly map the linear peptide epitopes targeted by human antibody responses to a pathogen.

## 2. Materials and Methods

### 2.1. Patient Serum Samples

DENV-positive serum samples used in this study were previously described [2]. Patient serum was obtained from DENV-infected individuals seven days post the onset of fever. Samples were defined as primary infection (IgM positive/IgG negative) or secondary infection (IgM and IgG positive) using the DENV IgG capture ELISA and PanBio Dengue IgM capture ELISA (Alere, INC.). DENV-negative (control) sera were collected from individuals who identified as never having been infected with DENV and did not live in a DENV endemic region. Institutional Review Board approval was granted by both institutions involved in this study (Bioethics Research Committee of the Gorgas Memorial Institute for Health Studies and the UNMHSC/School of Medicine IRB Committee).

### 2.2. Deep Sequence-Coupled Biopanning (DSCB)

The method used for the deep sequence-coupled biopanning has been previously described [1,2,27] and had the following modifications. IgG was isolated from 20 µL of patient sera using Dynabeads Protein G (Invitrogen) following the manufacturer’s instructions. The concentration of IgG was then estimated by absorbance at 280 nm as measured by Nanodrop. IgG (500 µg) was mixed with 10 μg of DENV-3 antigen fragment library displayed on MS2-VLPs and brought to a total volume of 100 µL with phosphate-buffered saline (PBS) [2]. This was incubated overnight at 4 °C with gentle agitation. VLP/antibody complexes were pulled down using 10 μL of Dynabeads Protein G with incubation at 4 °C with shaking for 1 h. Complexes were then washed 6 times in 0.5% Tween PBS (PBS-T) and 3 times in PBS (200 µL volumes), transferring to fresh tubes after wash 1, 4, and 7. VLPs were then eluted from the Dynabeads with 50 µL 0.1 M glycine pH 2.7 for 5 min at room temperature and immediately neutralized with 5 µL 1 M tris pH 9.0. RNeasy Micro Kit was used to isolate and purify nucleic acid from eluted VLPs. Eluted VLP RNA was then used as template in RT-PCR using the following conditions: 4.0 µL E2 primer (5′-TCAGCGGTGGCAGCAGCCAA-3′) with 1.0 µL 10 mM dNTP mix (Invitrogen) and 8 µL eluted VLP RNA heated to 65 °C for 5 min and quick chilled on ice. Next, 4.0 µL of 5X First Strand Buffer (Invitrogen) and 2.0 µL 0.1 M dithiothreitol were added and heated to 37 °C for 2 min. Finally, 1 µL Superscript II Reverse Transcriptase (Superscript II, Invitrogen) was added and the reaction was incubated at 37 °C for 50 min followed by 15 min at 70 °C. Reverse transcriptase-reaction (1 µL) was used in PCR with 5 µL 10X PCR buffer, 2 µL MgSO_4_, 1 µL 10 mM dNTP mix (Invitrogen), 2.5 µL 62 up primer (5′-CTATGCAGGGGTTGTTGAAG-3′), E3.2 primer (5′-CGGGCTTTGTTAGCAGCCGG-3′), and 0.2 µL Platinum HiFi Taq polymerase (Invitrogen) in a 50 µL total reaction volume. The mixture was placed in a thermocycler and cycled at the following specifications: 94 °C for 2 min, 30 cycles of (94 °C for 30 s, 60 °C for 30 s, 68 °C for 30 s), 68 °C for 10 min, and 4 °C hold. PCR product was confirmed by agarose gel electrophoresis and then purified with QIAquick PCR Purification kit (Qiagen) using the manufacturer’s instructions. DNA was then used as a template for 15 cycles of PCR with Ion Torrent-barcoded primers. PCR products were then run on a 1.2% agarose gel in Tris-Borate-EDTA buffer (45 mM Tris-Borate, 1 mM EDTA), excised with a scalpel, and extracted using QIAquick Gel Extraction kit (Qiagen) following the manufacturer’s instructions. The purified PCR product was then subjected to Ion Torrent deep sequencing. Raw data were processed with custom MATLAB scripts as previously described [1,2,27]. Sequences that passed quality control standards were then used to identify sequences encoding unique peptides and rank them according to their abundance (% total population of quality-controlled sequences). The starting DENV-3 antigen fragment plasmid library was also subjected to Ion Torrent deep sequencing and used to determine the fold enrichment of each peptide (% population sequences for patient biopanning/% population sequences of starting library). For peptides identified in patient biopanning samples but not present in the starting library, we assumed an abundance of 1 read in order to calculate fold enrichment. Duplicate samples used the same isolation of IgG from sera but were prepared as independent samples after this point.

### 2.3. Structure and Alignments

Utilizing National Center for Biotechnology Information (NCBI) BLAST against the reference sequence for dengue virus 3 polyprotein (accession #: YP_001621843.1), alignments of the DENV polyprotein were created.

### 2.4. Synthetic Peptide ELISA

Synthetic peptide ELISAs were performed as previously described [2] with the following modifications. Immunolon 96-well ELISA plates were coated with 1 µg/100 µL of streptavidin (Invitrogen) in PBS and incubated overnight in 4 °C. Plates were washed 3 times with PBS and 2 µg/100 µL of succinimidyl 6 ((beta-maleimidopropionamido) hexanoate) heterobifunctional (SMPH) crosslinker was added and incubated for 1 h at room temperature with rocking. Plates were washed 3 times with PBS and custom synthetic peptides containing a C-terminal cysteine (GenScript) were added in 100 µL volumes at 0.02 µg/µL and incubated at room temperature for 2 h with rocking. Plates were then washed 3 times with PBS and blocked over night with 300 µL of 0.5% dry milk in PBS containing 0.05% Tween 20 (PBST) (Bio-Rad) at 4 °C. Blocking solution was removed, and plates were washed two times with PBST. Patient sera were diluted in 0.5% milk/PBST and added to 96-well plates in 100 µL volumes at the following dilutions: 1:20,000 for Fusion Loop (E 96–118) and 1:320 for NS1 56–71, NS1 154–170, NS1 193–204, and NS1 325–337. Sera were incubated for 2 h at room temperature with rocking and wells were washed 5 times with PBST. Goat anti-human IgG (Jackson ImmunoResearch) conjugated with horseradish peroxidase was then added in 100 µL volumes at 1:5000 dilution in 0.5% milk/PBST and incubated for 1 h at room temperature with shaking. Plates were then washed 5 times with PBST, followed by 5 washes with PBS. Next, 100 µL of soluble 3,3′,5,5′-Tetramethylbenzidine (TMB) (Millipore Corp.) developing reagent was added to each well, and plates were incubated at RT with shaking until sufficiently developed (15 min) and quenched with 100 µL of a 1% HCl solution, and absorbance at 450 nm was determined.

### 2.5. MATLAB Scripts and Statistical Analysis

Statistical analyses of data were carried out using PRISM 8 for Macintosh. Statistical analysis investigating differences between DENV-infected patient sera and negative patient sera via peptide ELISA was conducted using a Kruskal–Wallis test. Correlation of mean fold-change versus ELISA readout (ABS 450) was analyzed using a Spearman r correlation test. Values were deemed statistically significant with a *p*-value ≤0.05. MATLAB scripts used for analysis were previously described and are available upon request from the corresponding author [1,2,27].

## 3. Results

### 3.1. Modifications to DSCB Protocol

**Figure 1:**
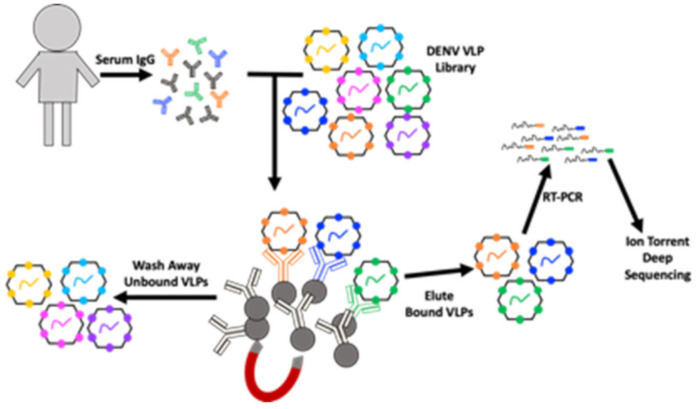
Schematic showing the workflow for our modified deep sequence-coupled biopanning (DSCB) approach. IgG was isolated from human serum and mixed with an MS2 virus-like particle (VLP) library displaying all possible 10-amino acid peptides from the dengue virus (DENV) polypeptide. Protein-G coupled to magnetic beads are used to pull down antibody–VLP complexes. Bound VLPs are eluted, RNA is extracted, and RT-PCR is used to produce cDNA, which is then subjected to Ion Torrent deep sequencing.

DSCB is a method for identifying the peptide targets of antibodies in a polyclonal mixture (e.g., serum) (Figure 1). We previously used DSCB to investigate the specificity of antibodies in serum from patients infected with DENV [2]. As in that work, here we used a DENV-VLP library constructed from MS2 VLPs. This library was produced by site-directed mutagenesis of the MS2 coat protein in pDSP62 plasmid as previously described [2,4,28]. The DENV-VLP library contains all possible 10 amino acid peptides displayed on the surface of the MS2-VLP in a constrained beta-hairpin called the AB loop. IgG isolated from patient sera was mixed with the DENV-VLP library, unbound VLPs were washed away and VLPs were eluted off of the antibodies. Our previous use of DSCB required cloning of the affinity-selected, RT-PCR-amplified sequences in preparation for deep sequence analysis, which allowed for only 2–10 samples to be prepared and analyzed in one week [1,2]. However, we sought to streamline our previously published process by preparing the RT-PCR product directly for sequencing, eliminating the bottleneck created by molecular cloning (Figure 1). This approach allows us to test up to 60 samples in the same time frame (1 week) as our previously published work. We then performed DSCB in this way on dengue virus primary infection patient samples (n = 28). Each sample was processed in duplicate and then analyzed with our custom MATLAB scripts. Two of the 28 primary samples did not return a usable duplicate, and those two samples were removed from further analysis. Analysis of individual patient results (showing both replicates) is shown in supplementary figures (Supplementary Figures S1–S26, panels A, B, and C).

### 3.2. Comparison of Sera from 26 Primary DENV- Infected Individuals for Commonly Selected DENV-NS1 and Envelope Epitopes

**Figure 2:**
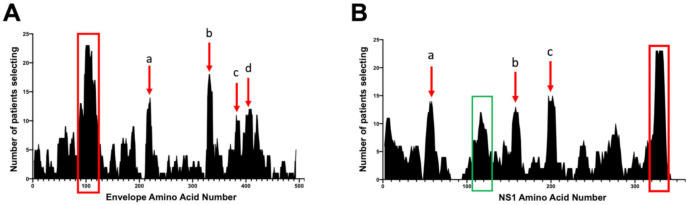
Comparison of 26 primary infected sera samples to determine population-wide antibody responses. Sequence analysis among the 26 serum samples were then combined to compare the number of study subjects selecting for specific regions at a pre-determined fold cutoff (>100-fold enrichment compared to the starting library) of envelope (E) polypeptide (**A**), or non-structural protein 1 (NS1) polypeptide (**B**). Red boxes represent regions selected by more than 75% of the serum samples. Arrows indicate other regions that were selected by ~50% of the study subjects. (**A**) E protein in domain II (arrow a), domain III (arrow b and c), and the juxtamembrane stem region (arrow d). (**B**) NS1 56–71 arrow a, 154–170 arrow b, and 193–204 arrow c. The green box indicates the previously described wing domain (WD).

We next investigated common DENV epitopes targeted by human antibody responses. As expected, human antibodies targeted previously reported immunodominant epitopes of DENV envelope (E) and non-structural protein 1 (NS1) (Figure 2). Since antibodies against E and NS1 have been implicated in both protection from infection and enhanced pathogenesis, we focused on antibody responses against these two viral proteins. Figure 2 shows the number of patients with antibodies against epitopes that included specific amino acids in either E (Figure 2A) or NS1 (Figure 2B) (defined as a >100-fold enrichment compared to the starting VLP-DENV library).

The E protein, the primary antigenic structural protein of DENV, includes domain I (a.a. 1–51, 131–191, and 271–298), domain II (a.a. 52–130 and 192–270), domain III (a.a. 299–393), the juxtamembrane stem region (a.a. 394–448), and the transmembrane domain (a.a. 449–495). For the E protein, 23 of the 25 patients selected peptides corresponding to the fusion loop region, (a.a. 96–118), indicated by boxed region (Figure 2A). Additionally, several other regions of the E protein were selected by at least half of patients (indicated by arrows). They map to E protein in domain II (Figure 2A, arrow a), to domain III (Figure 2A, arrow b and c), and the juxtamembrane stem region (Figure 2A, arrow d) [29].

The NS1 protein is a non-structural protein that exists as both a dimer and hexamer. Peptides in the NS1 tail region (a.a. 325–340, the red box in Figure 2B) were selected by 23 of the 25 patients. The NS1 tail region is a potential recognition site of non-neutralizing antibodies that may also bind to platelets, thus disrupting coagulation in DENV-infected individuals [25,30]. In contrast, the wing domain epitope (a.a. position 102–112), a previously identified immunodominant epitope, was selected by only 10 patients (Figure 2B, green box). Three other regions of NS1 (NS1 56–71 arrow a, 154–170 arrow b, and 193–204 arrow c) were also selected by over half of the patients (Figure 2B, red arrows).

**Figure 3:**
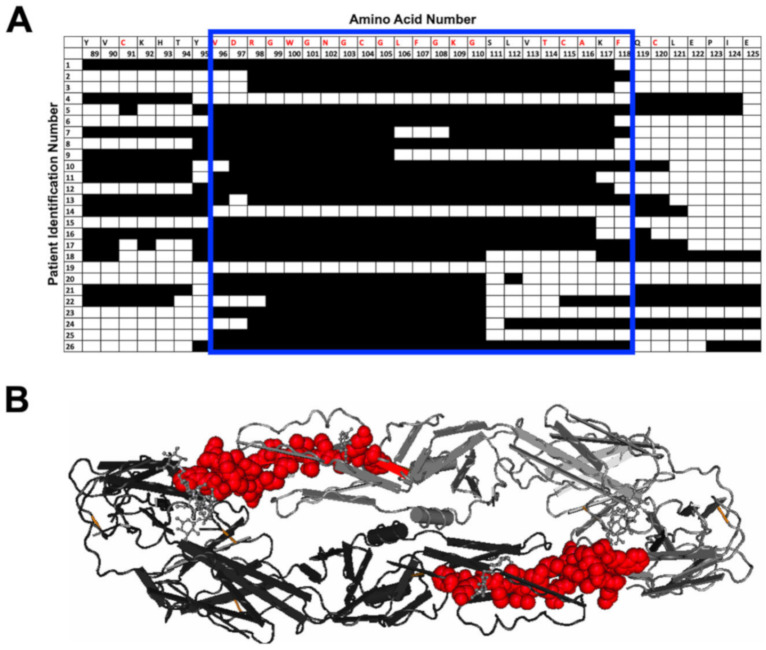
Individual amino acid selection for the fusion loop region. (**A**) Patterns of patient selection of amino acids of the wing domain of the E protein were analyzed for each individual amino acid position. Patients selecting for a specific amino acid were filled in (black), and if no selection was seen for the amino acid position, the box remained unfilled. Amino acids conserved among all 4 serotypes are written in red along the top of the table. The fusion loop has been highlighted by a blue box. (**B**) DENV envelope protein dimer is shown with red highlighting the fusion loop amino acids selected in our DSCB experiment.

**Figure 4:**
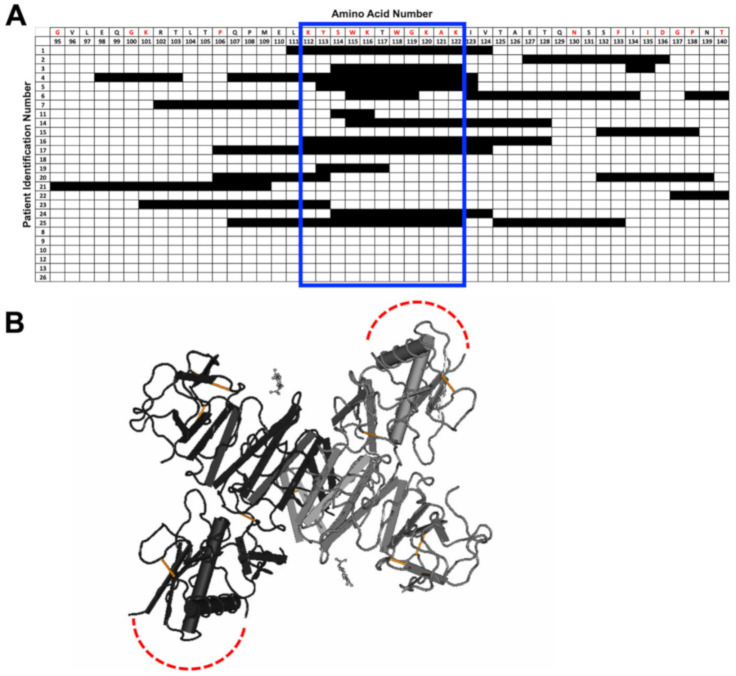
Individual amino acid selection for the NS1 wing domain. (**A**) Patterns of patient selection of amino acids of the wing domain of the NS1 protein were analyzed for each individual amino acid position. Patients selecting for a specific amino acid were filled in (black), and if no selection was seen for the amino acid position, the box remained unfilled. Amino acids that are conserved among all 4 serotypes of DENV are written in red along the top of the table. The regions that correlate to the wing domain have been highlighted by a blue box. (**B**) NS1 wing domain epitope is highlighted on the structure of the NS1 dimer in red (PDB: 4O6B). The wing domain is unresolved in the structure and is indicated by dotted red lines.

**Figure 5:**
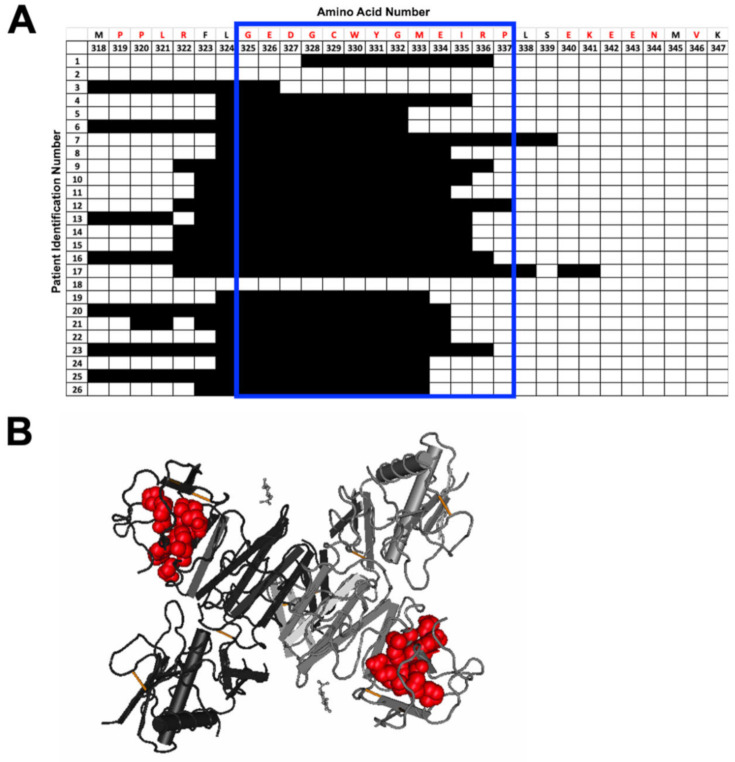
Individual amino acid selection for NS1 tail region. (**A**) Patterns of patient selection of amino acids of the tail region of the NS1 protein were analyzed for each individual amino acid position. Patients selecting for a specific amino acid were filled in (black), and if no selection was seen for the amino acid position, the box remained unfilled. Amino acids that are conserved among all 4 serotypes of DENV are written in red along the top of the table. The regions that correlate to the NS1 tail have been highlighted by a blue box. (**B**) NS1 tail region epitope is highlighted on the structure of the NS1 dimer in red (PDB: 4O6B).

Next, we more closely examined the specific amino acids selected by each individual in several regions of specific interest: the E protein fusion loop (Figure 3), the NS1 wing domain (Figure 4), and the NS1 tail region (Figure 5). The fusion loop (a.a. 96–117) was commonly selected among the 26 patient samples. In contrast, we observed considerable diversity in the specific peptides selected within the NS1 wing domain among patients (Figure 4). Several patients did not select any epitopes within the wing domain (patients 8–10, 12, 13, and 26). Most sera (23/26) selected peptides from the highly conserved NS1 tail, the region spanning a.a. 325–337 (Figure 5). Two patients failed to select any epitopes in this region.

### 3.3. Peptide-Specific Antibody Responses to Commonly Selected Epitopes within NS1 and E

**Figure 6:**
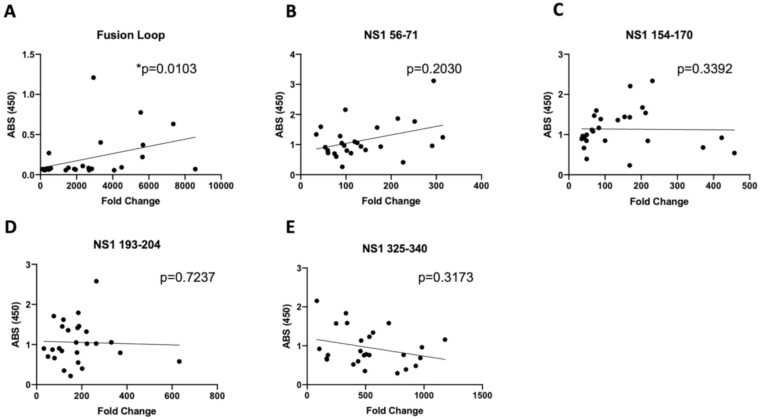
Correlation of DSCB fold change and corresponding ELISA readings. ELISA absorbance was compared to the average of the highest fold change values from the DSCB data for (**A**) Fusion loop, (**B**) NS1 56-71, (**C**) NS1 154-170, (**D**) NS1 193-204, and (**E**) NS1 325-340. *p*-values of correlation were determined using a Spearman correlation test, and linear regression lines are shown.

To further investigate the utility of DSCB as an epitope discovery tool, we sought to determine the serum binding activity for individual peptides and investigate whether the fold-enrichment of peptides was predictive of the magnitude of the antibody response. Using the serum samples screened by DSCB, we performed ELISA against a subset of peptides of interest and looked for a correlation with the enrichment (fold change) of that peptide family for each sample (Figure 6). The regions we targeted in this analysis included two that were strongly selected (the fusion loop peptide (E 96–118) and the NS1 tail (NS1 325–340)) and three regions of NS1 that were selected by a subset of patients (NS1 56–71, NS1 154–170, and NS1 193–204) (Figure 6). Peptide ELISAs for the wing domain were reported in previous work with these samples and showed no significant difference between primary- and secondary-infected individuals [2]. Although we observed a statistically significant correlation between the fold enrichment and ELISA reading for the E fusion loop (Figure 6A), this correlation was not striking. The fusion loop in particular was the most enriched peptide region for all patient sera for which we performed DSCB (Supplementary Figures S1–S26). The lack of correlation between ELISA and fold enrichment suggests that the DSCB technology may not provide quantitative information on the magnitude of antibody responses as measured by ELISA.

**Figure 7:**
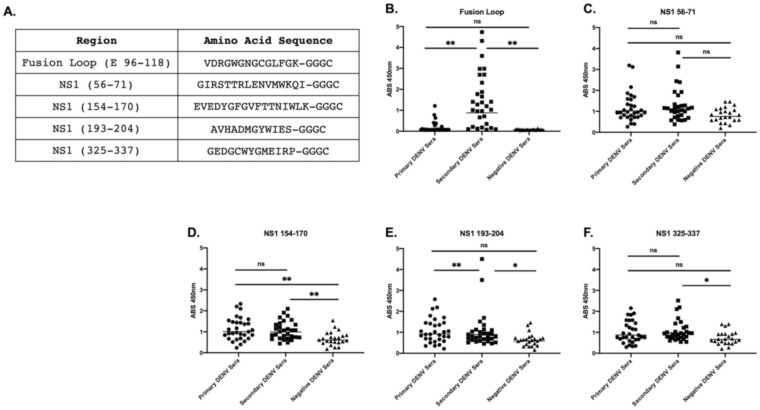
Five highly selected peptides were tested via IgG peptide ELISA. Five sequences were identified as highly selected among the 25 primary infected patients, and a terminal -GGGC was added to the peptides for the attachment of peptide to the ELISA plates (**A**). Sera from primary and secondary DENV-infected patients, as well as negative controls were then tested against peptide ELISA to identify whether sera bound to regions observed from the deep sequence-coupled biopanning results. Fusion loop peptide (**B**), NS1 56–71 (**C**), NS1 154–170 (**D**), NS1 193–204 (**E**), NS1 325–337 (**F**). * Indicates *p* < 0.05, ** *p* < 0.005 as determined by Kruskal–Wallis test of multiple comparisons.

Next, we investigated additional sera samples from patients with secondary infections as well as sera from DENV-negative individuals as negative controls. Primary and secondary DENV infections have been shown to have differences in antibody specificity, so we were interested to investigate the peptide-specific antibody responses we identified by DSCB in primary samples in secondary DENV samples. Sera from secondary infections showed reactivity to the fusion loop (E 96–118) and the NS1 tail (NS1 193–204) compared to both primary and negative patient sera (Figure 7B,E). These sera had significant reactivity to NS1 325–337 (Figure 7F), but no more than sera from primary infections. For NS1 154–170, sera from primary and secondary infections had significantly higher reaction than control patients but were not significantly different from each other (Figure 7D). We did not observe significant differences in antibody reactivity among the groups for NS1 region 56–71 (Figure 7C).

## 4. Discussion

DENV continues to be a global health threat to millions of people each year, yet a safe vaccine for population-based immunization strategies does not currently exist. The discovery of tools to identify immunogenic epitopes from pathogens continues to be a useful contribution to vaccine development. Identification of DENV epitopes through the use of short peptides has been the most common method used thus far for exploring the antibody profiles induced by DENV. These methods have been employed using both mouse and human sera [31,32,33]. However, these investigations have focused mostly on the envelope protein, with few studies of antibody profiles of the entire DENV genome [34,35,36]. Here, we utilized our DSCB technique and modified it to streamline the approach and make this a more efficient technology for rapid antigen discovery.

Our technique has identified a number of interesting targets in individuals with primary DENV infection. Indeed, the epitopes we identify here are largely comparable to our previously reported use of DSCB on DENV patient samples using our old workflow [2]. These targets include not only regions within DENV E such as the fusion loop but also multiple regions of NS1 that include, but are not limited, to the immunodominant wing domain. In addition to these well-described immunodominant epitopes, we have also identified additional regions targeted during DENV infection. Structure–function studies of DENV NS1 have shown that the wing domain and tail region are important targets of antibodies that cross-react with host proteins and may play a role in pathogenesis [25,30]. The importance of the other NS1 epitopes identified here by DSCB is unknown, but additional studies should investigate the potential for these antibodies to neutralize NS1 activity and/or cross-react with host proteins similar to other NS1 antibodies.

Our identification of antibodies against the E protein fusion loop is consistent with previous work utilizing alanine scanning analysis of predicted surface-exposed E protein epitopes, E protein epitope-specific knockout DENV VLPs, and Western blot analysis of serum antibodies [37,38,39,40,41]. Fusion loop antibodies are common in DENV-infected patients. Interestingly, the E protein fusion loop was both commonly selected by DENV-primary-infected patients but also showed the most enrichment (fold change) compared to other peptides. This suggests that the E protein fusion loop is an immunodominant epitope, which has been indicated in other studies [37,38,39,40,41]. Additionally, the magnitude of the fusion loop antibodies is higher in the sera of DENV patients with secondary infection compared to that in those with primary infection.

The work described here further confirms that DSCB is a useful tool for antigen discovery using human sera samples. However, as is true with comparable screening tools, epitopes identified with DSCB must be confirmed by ELISA, and the magnitude of the antibody responses is not necessarily predicted by DSCB. For instance, the magnitude of antibody response to fusion loop was correlated with the DSCB-identified enrichment for fusion loop (Figure 6A), but this was not true for other peptides we investigated, and the correlation was not particularly striking. Therefore, although our DSCB is a useful qualitative tool for identifying antibody profiles for further investigation, its usefulness in identifying quantitative differences in antibody responses may be limited. Another possibility is that our peptide ELISAs display the peptide epitopes in a linear format, while the peptides displayed on the MS2 VLPs are in a constrained loop. This may account for a discrepancy for some peptides showing low ELISA readings but strong selection in DSCB. Taken together, DSCB as modified in this report allows for the fine mapping of pathogen-specific epitopes in an efficient and rapid manner. We also have used this technology to investigate antibody responses to *Chlamydia trachomatis*, identifying known immunodominant epitopes [3]. With these modifications, our technology may be helpful in quickly identifying natural antibody responses to emerging infectious diseases that could assist in the efforts toward rapid vaccine development.

## Supplementary Materials

The following are available online at https://www.mdpi.com/1999-4915/12/10/1114/s1, Supplementary Figures S1–S26.
