# Supplementary material for: Expansion and Refinement of Deep Sequence-Coupled Biopanning Technology for Epitope-Specific Antibody Responses in Human Serum
Source: Viruses. 2020 Sep 30;12(10):1114. doi: 10.3390/v12101114 (PMC7600589; doi:10.3390/v12101114)
Supplement: Supplementary file 1 [file viruses-12-01114-s001.pdf]

Patient 1

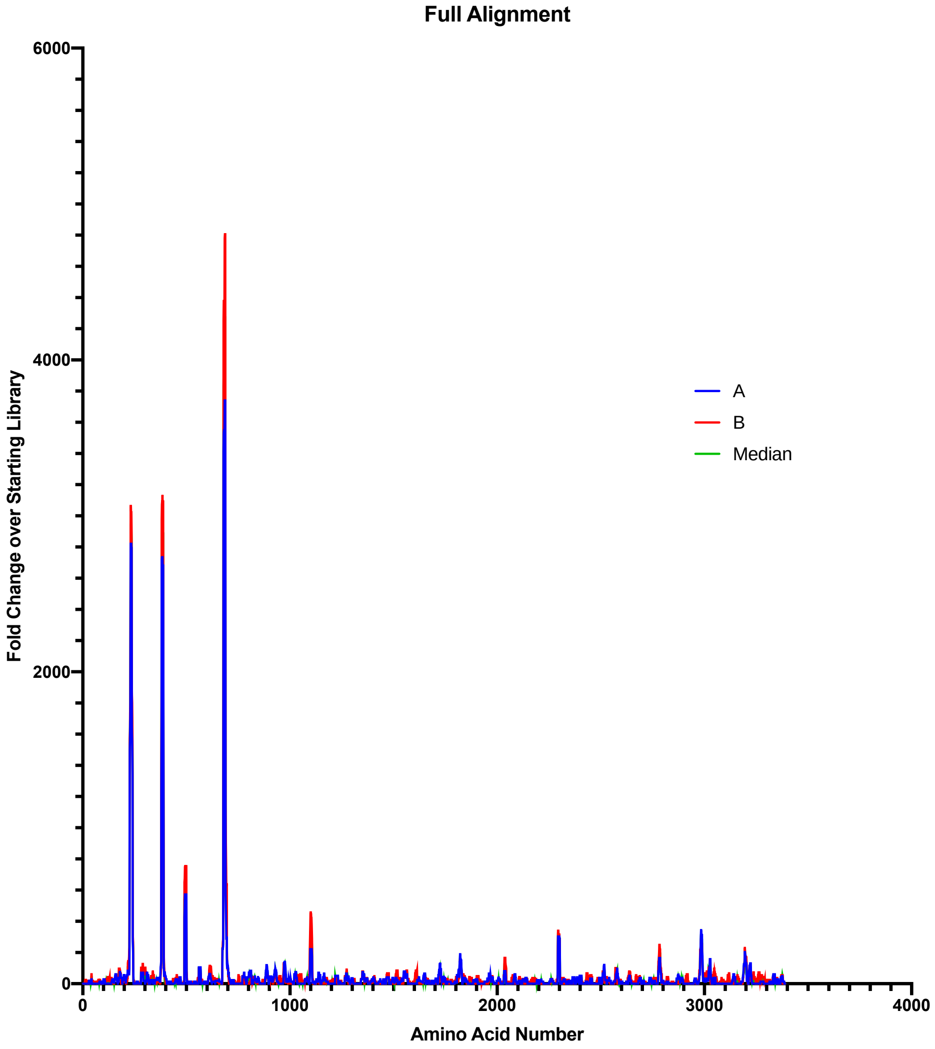
Full Alignment
Fold Change over Starting Library
6000
4000
2000
0
0
1000
2000
3000
4000
Amino Acid Number
A
B
Median

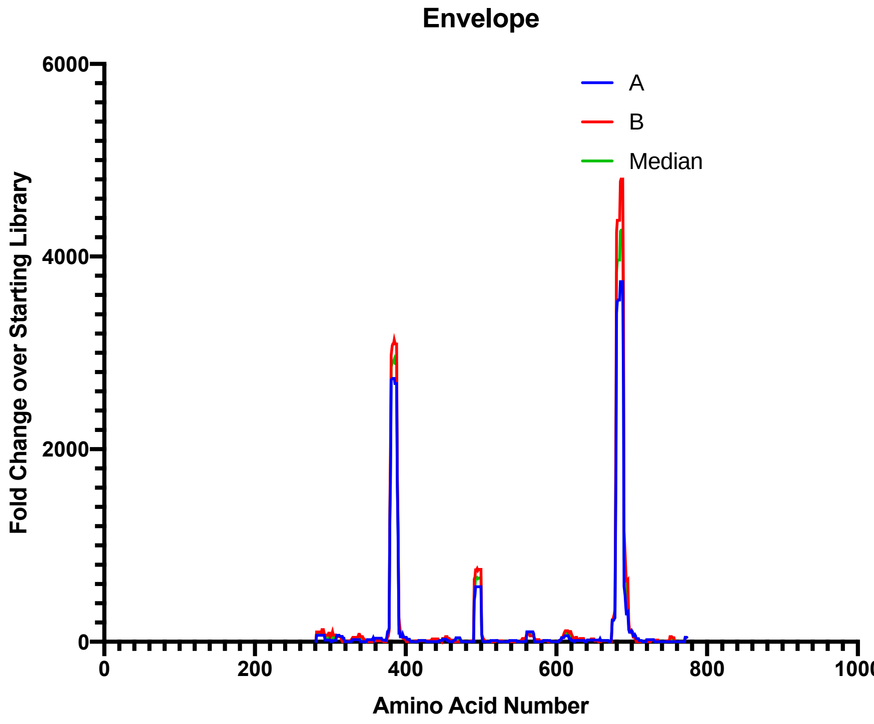
Envelope
Fold Change over Starting Library
6000
4000
2000
0
0
200
400
600
800
1000
Amino Acid Number
A
B
Median

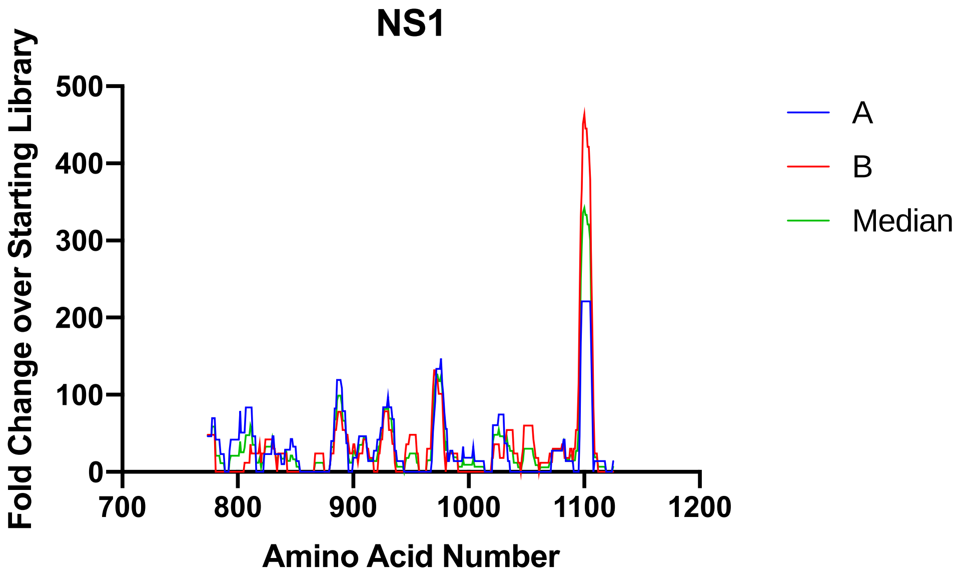
NS1
Fold Change over Starting Library
500
400
300
200
100
0
700
800
900
1000
1100
1200
Amino Acid Number
A
B
Median

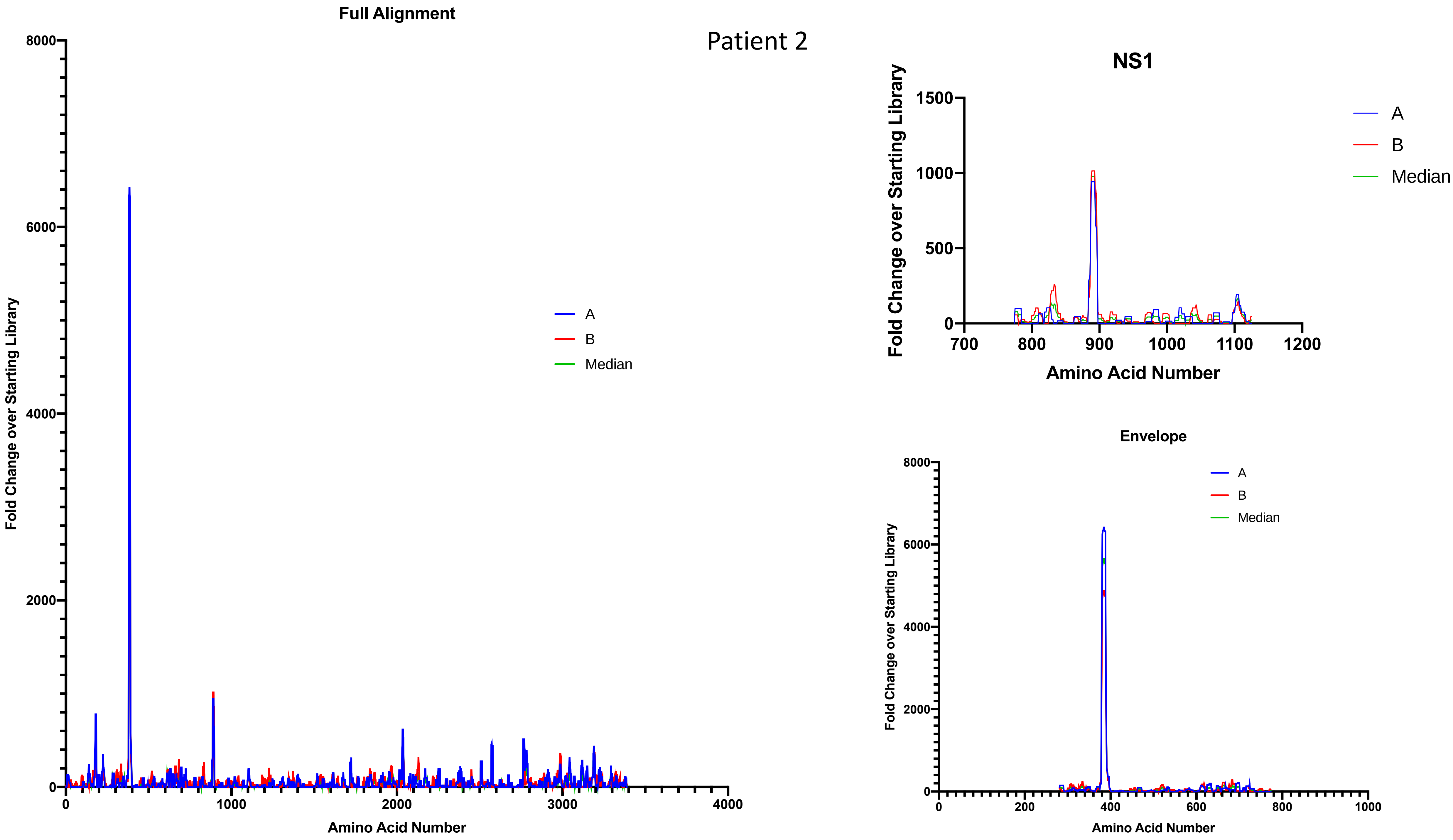
Patient 2
Full Alignment
Fold Change over Starting Library
Amino Acid Number
0
2000
4000
6000
8000
0
1000
2000
3000
4000
A
B
Median
NS1
Fold Change over Starting Library
Amino Acid Number
0
500
1000
1500
700
800
900
1000
1100
1200
A
B
Median
Envelope
Fold Change over Starting Library
Amino Acid Number
0
2000
4000
6000
8000
0
200
400
600
800
1000
A
B
Median

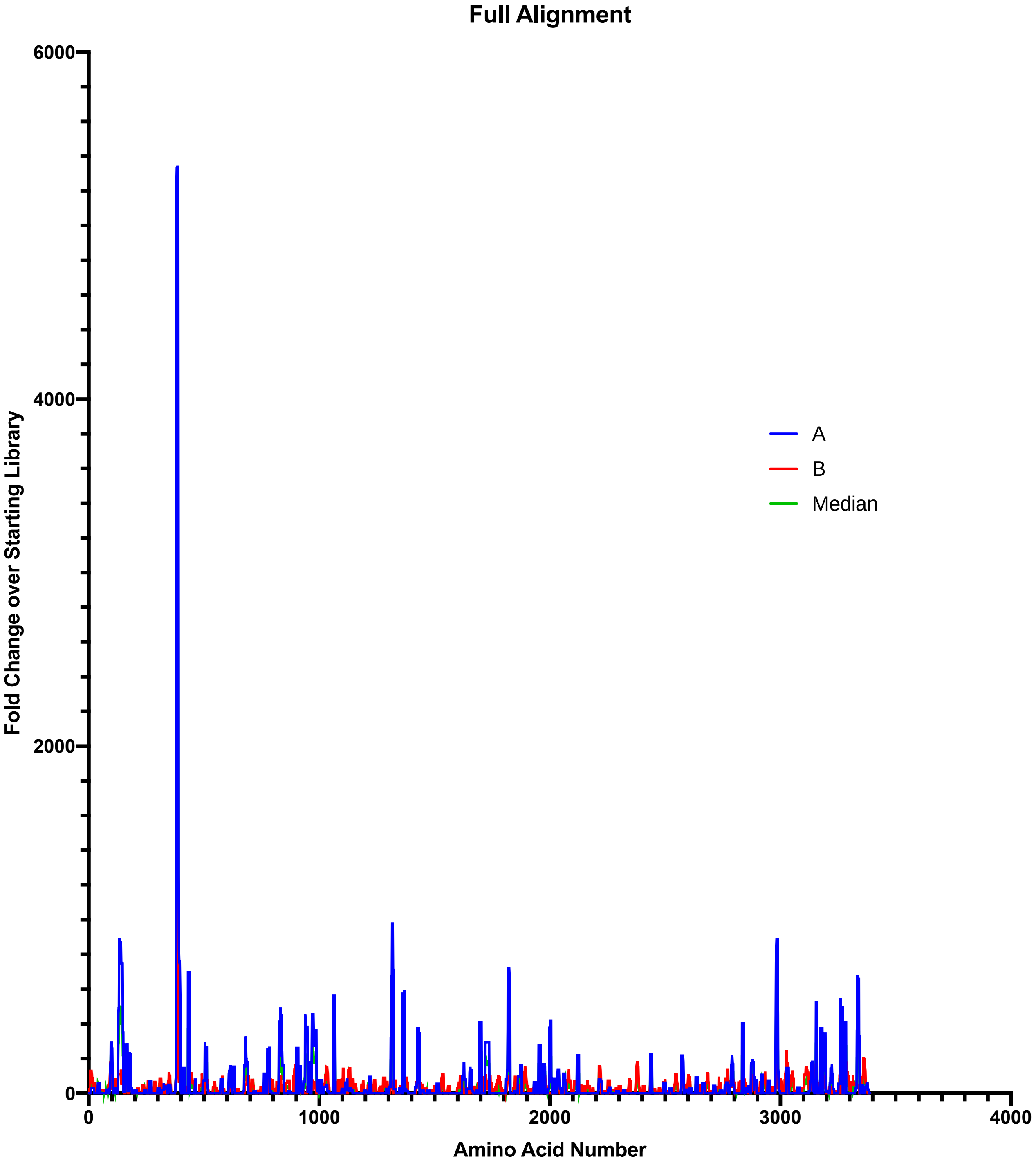
Full Alignment
Fold Change over Starting Library
Amino Acid Number
A
B
Median

Patient 3

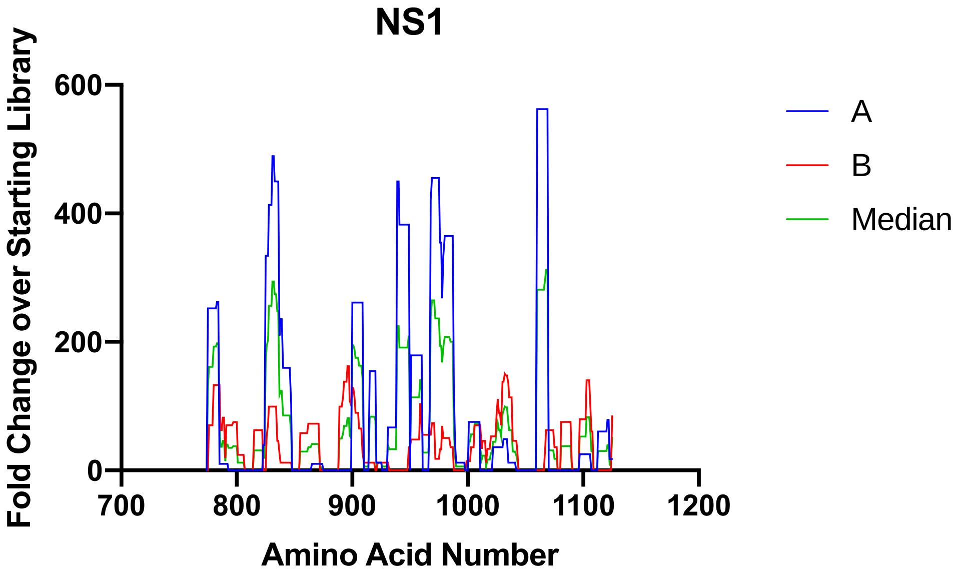
NS1
Fold Change over Starting Library
Amino Acid Number
A
B
Median

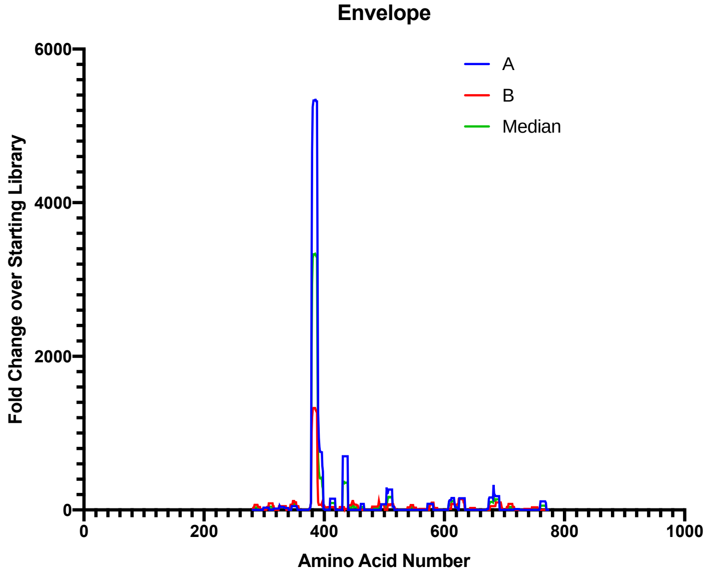
Envelope
Fold Change over Starting Library
Amino Acid Number
A
B
Median

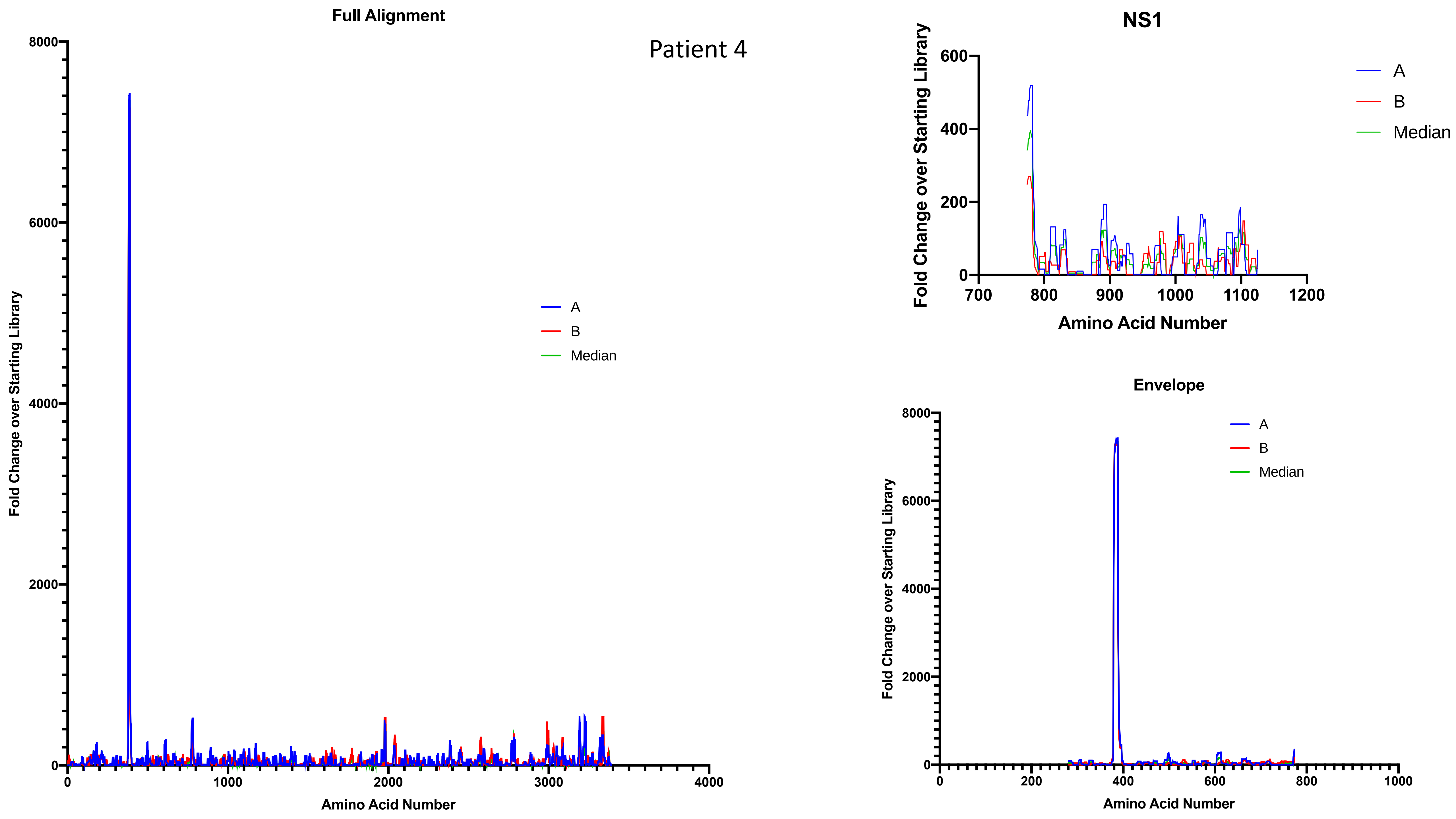

Full Alignment
Patient 4
8000
6000
4000
2000
0
Fold Change over Starting Library
0
1000
2000
3000
4000
Amino Acid Number
A
B
Median
NS1
600
400
200
0
Fold Change over Starting Library
700
800
900
1000
1100
1200
Amino Acid Number
A
B
Median
Envelope
8000
6000
4000
2000
0
Fold Change over Starting Library
0
200
400
600
800
1000
Amino Acid Number
A
B
Median

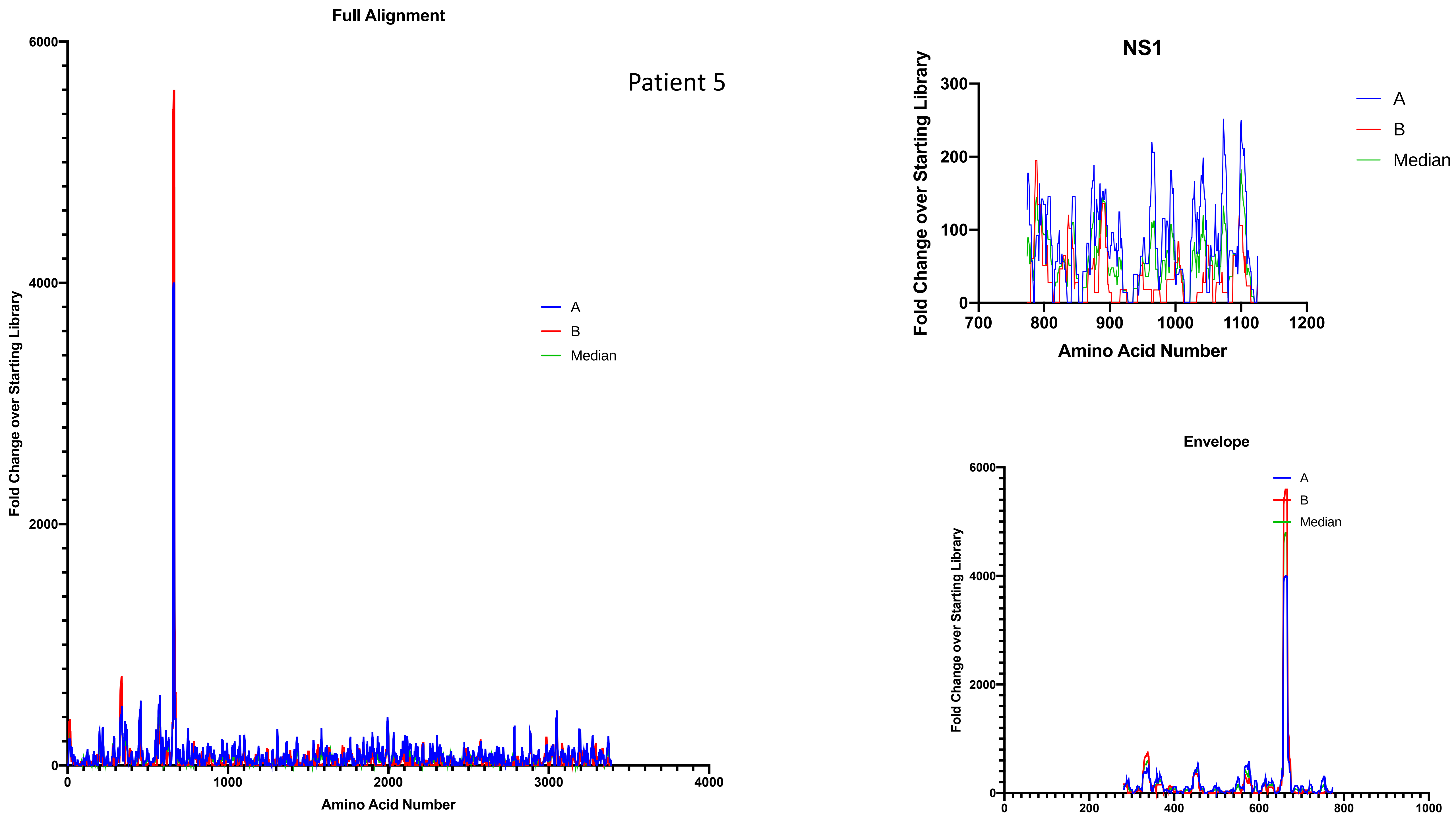
Full Alignment
Patient 5
Fold Change over Starting Library
Amino Acid Number
A
B
Median
0
2000
4000
6000
0
1000
2000
3000
4000
NS1
Fold Change over Starting Library
Amino Acid Number
0
100
200
300
700
800
900
1000
1100
1200
A
B
Median
Envelope
Fold Change over Starting Library
0
2000
4000
6000
0
200
400
600
800
1000
A
B
Median

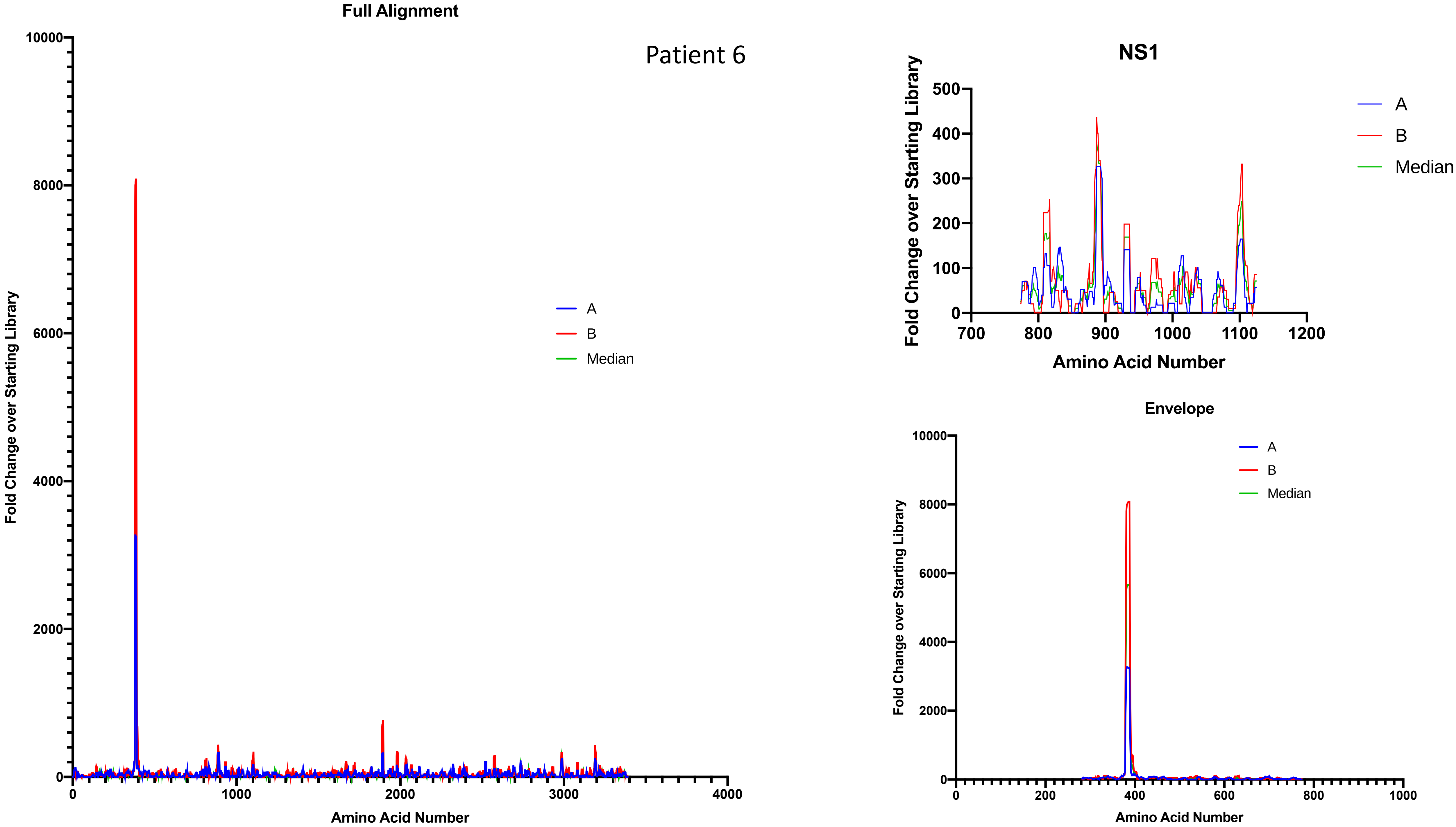

Patient 6
Full Alignment
Fold Change over Starting Library
10000
8000
6000
4000
2000
0
1000
2000
3000
4000
Amino Acid Number
A
B
Median
NS1
Fold Change over Starting Library
500
400
300
200
100
0
700
800
900
1000
1100
1200
Amino Acid Number
A
B
Median
Envelope
Fold Change over Starting Library
10000
8000
6000
4000
2000
0
200
400
600
800
1000
Amino Acid Number
A
B
Median

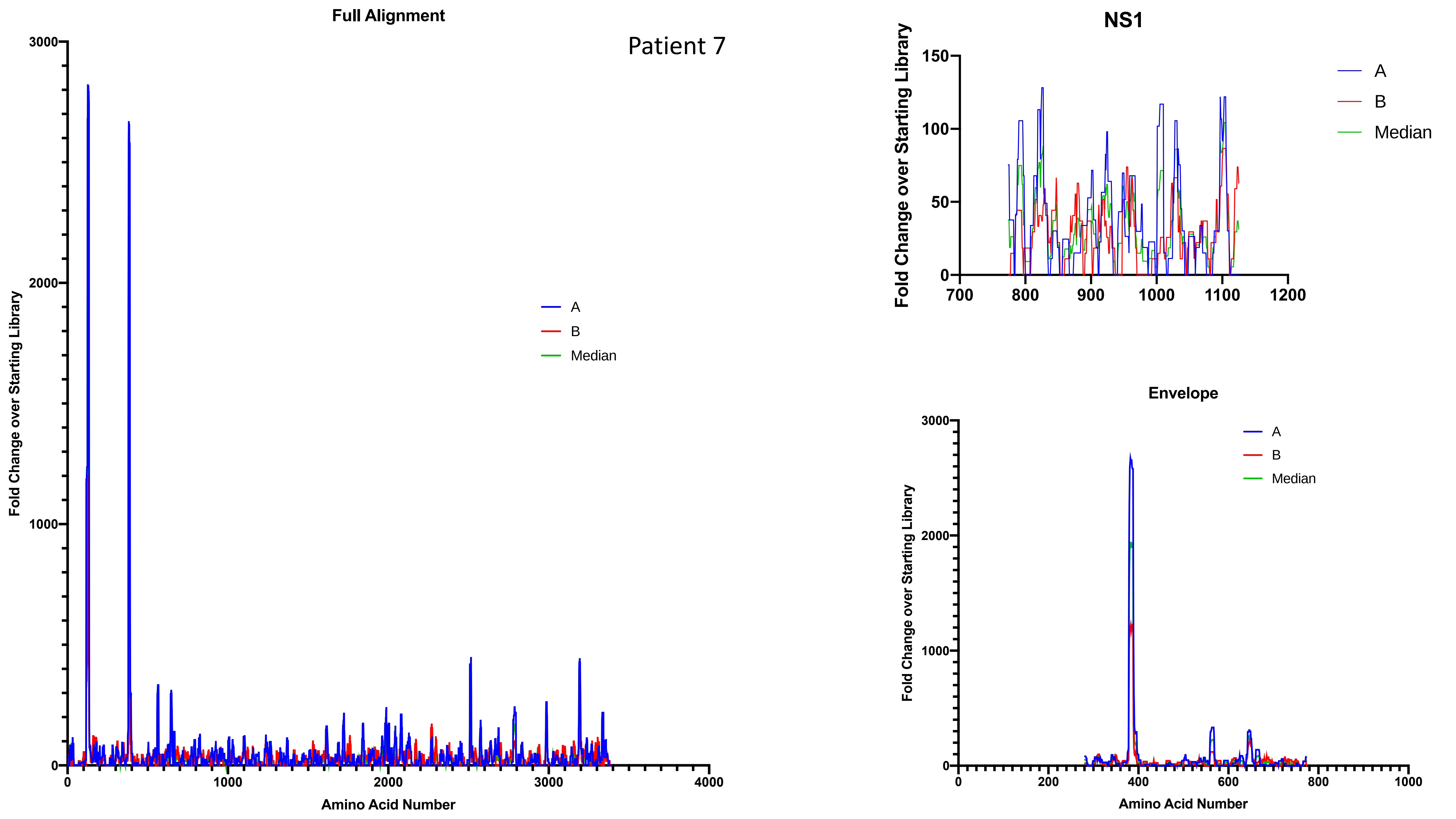
Full Alignment
Patient 7
Fold Change over Starting Library
Amino Acid Number
A
B
Median
NS1
Fold Change over Starting Library
A
B
Median
Envelope
Fold Change over Starting Library
Amino Acid Number
A
B
Median

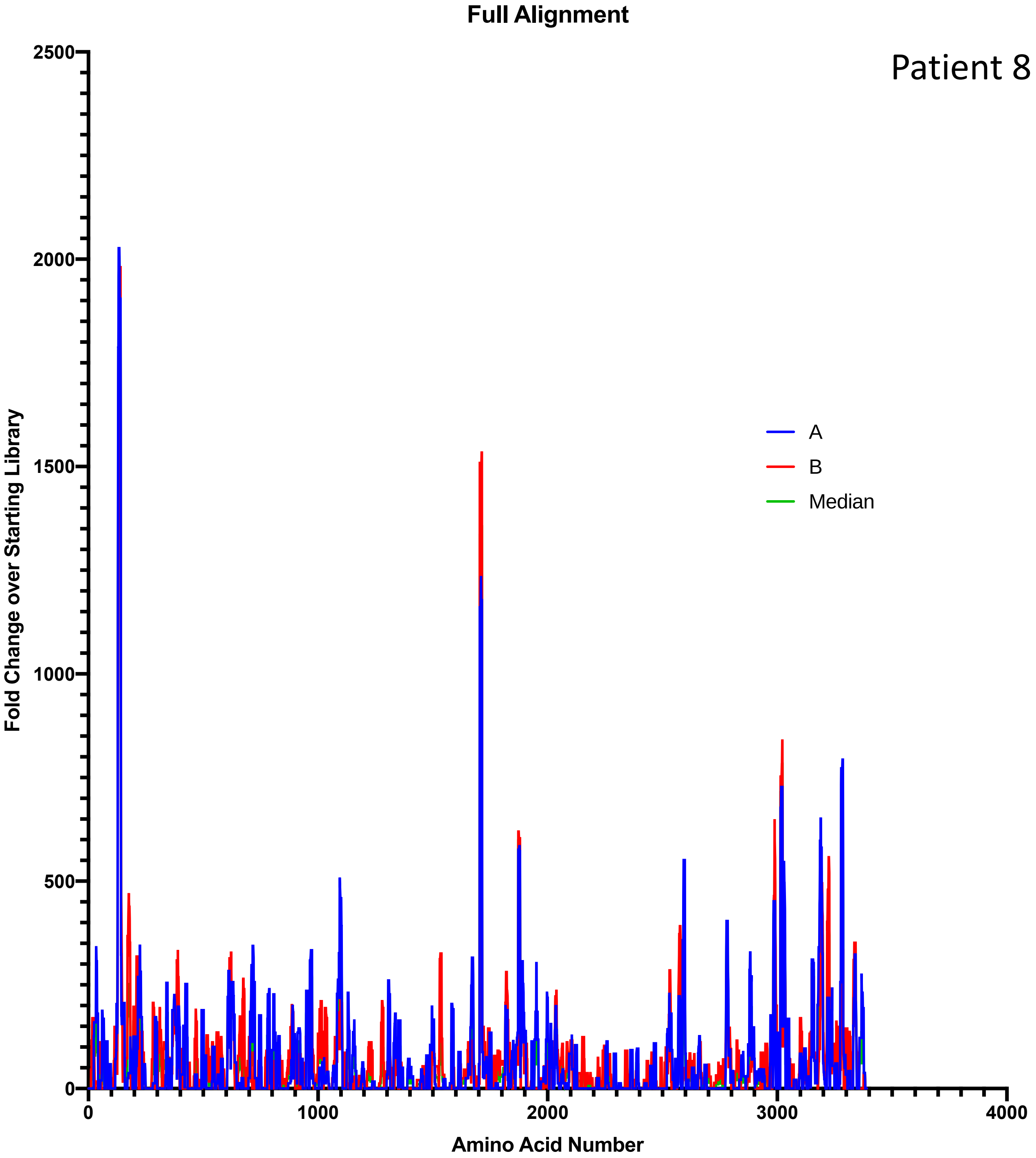
Full Alignment
Patient 8
Fold Change over Starting Library
2500
2000
1500
1000
500
0
0
1000
2000
3000
4000
Amino Acid Number
A
B
Median

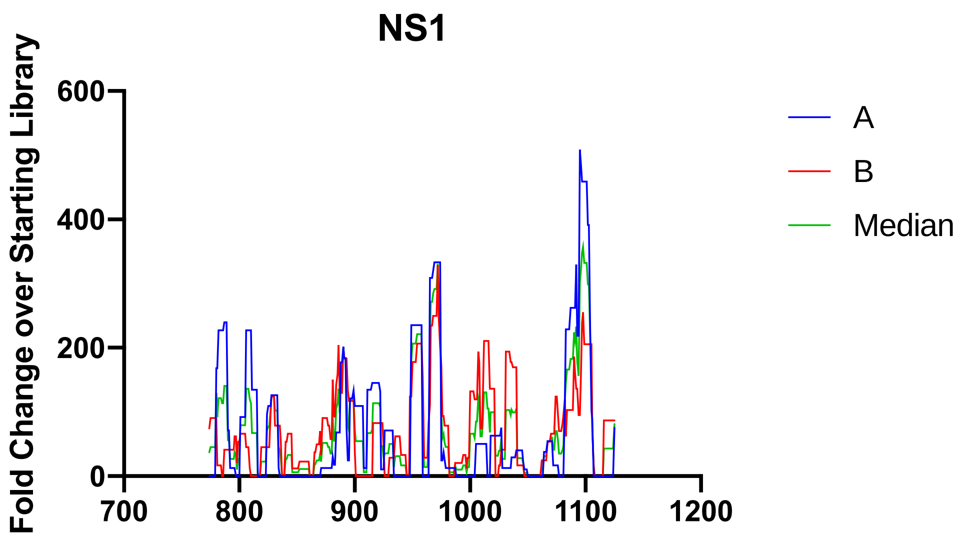
NS1
Fold Change over Starting Library
600
400
200
0
700
800
900
1000
1100
1200
A
B
Median

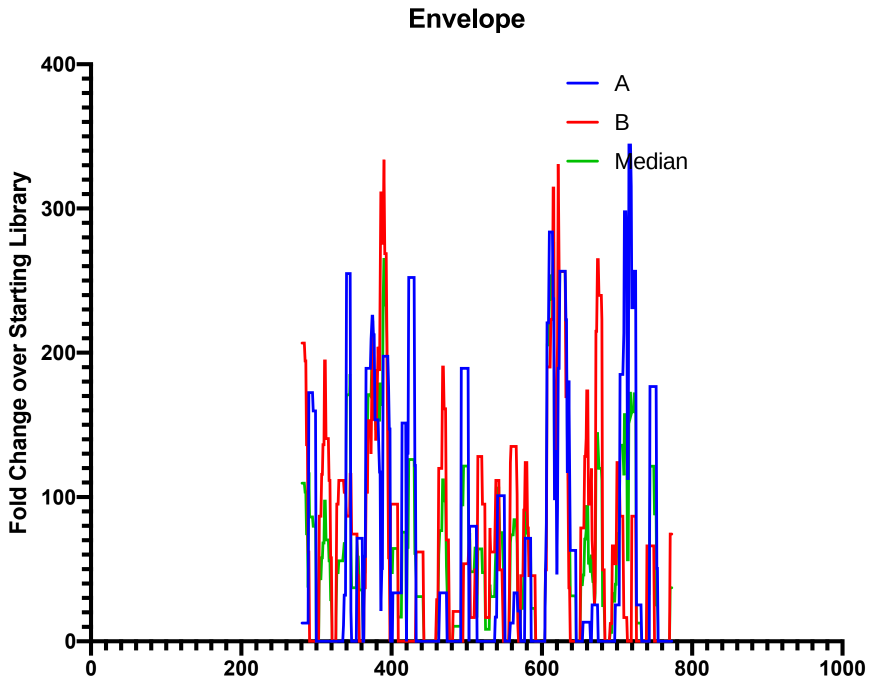
Envelope
Fold Change over Starting Library
400
300
200
100
0
0
200
400
600
800
1000
A
B
Median

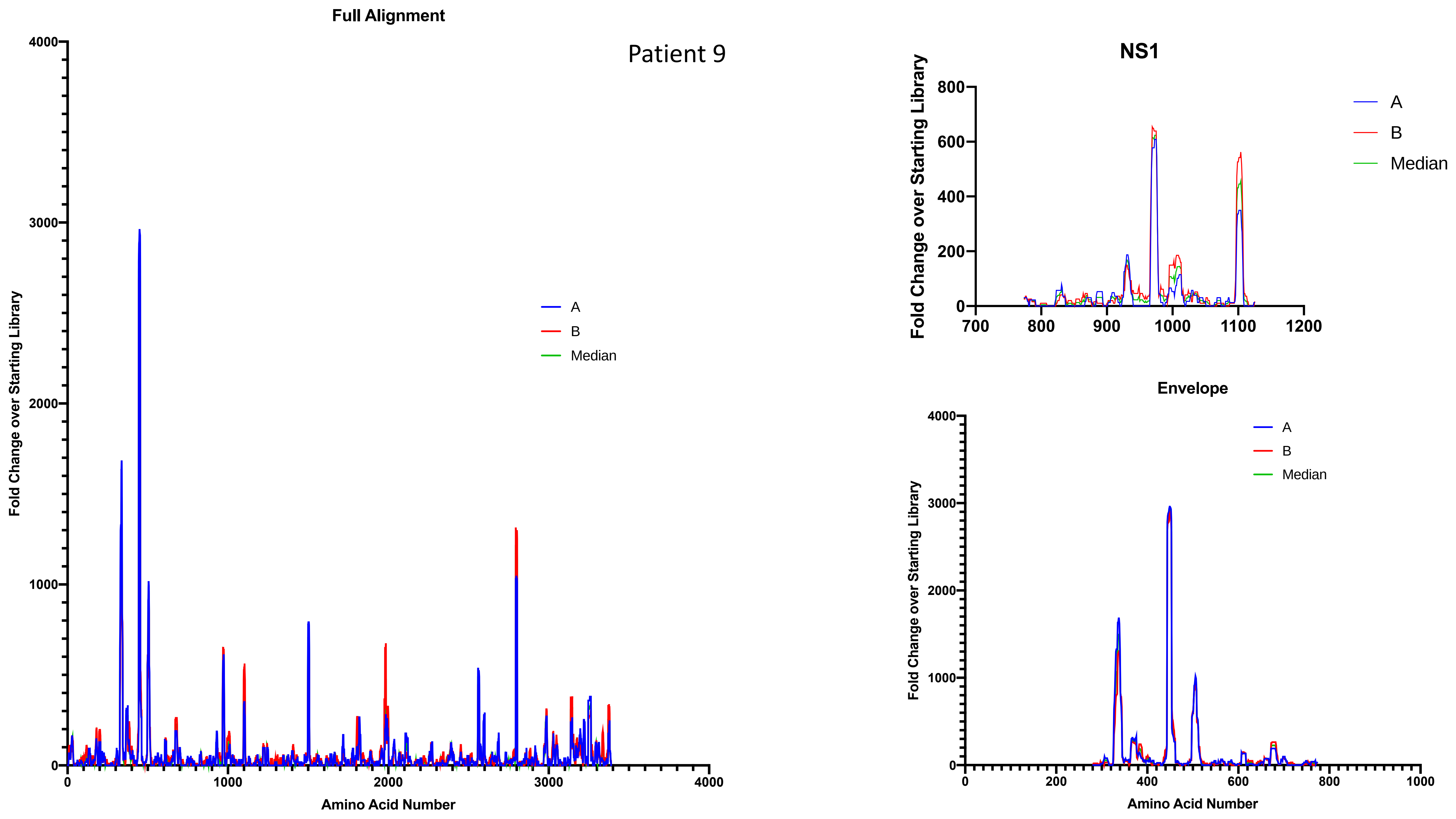
Full Alignment
Patient 9
Fold Change over Starting Library
Amino Acid Number
A
B
Median
NS1
Fold Change over Starting Library
A
B
Median
Envelope
Fold Change over Starting Library
Amino Acid Number
A
B
Median

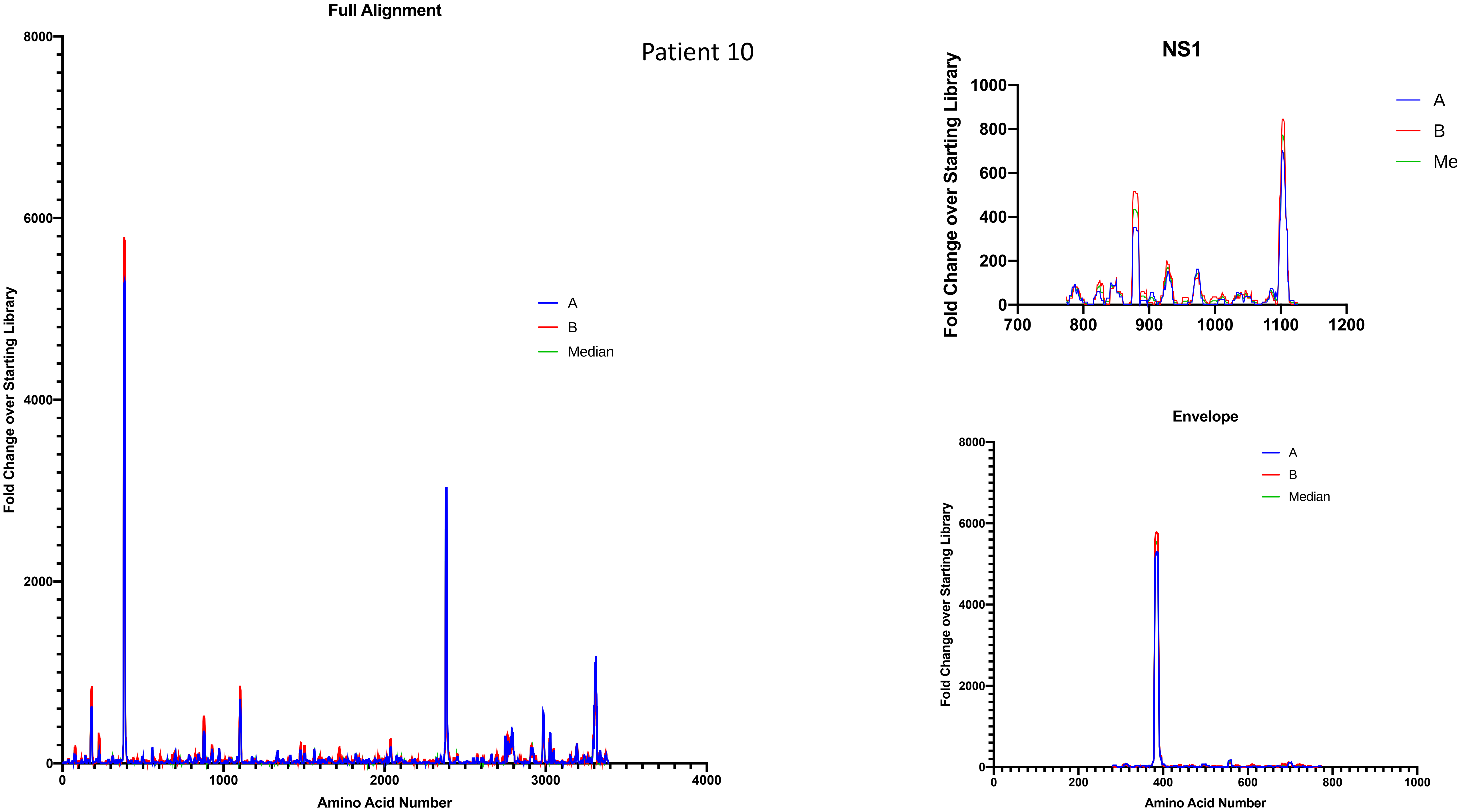
Full Alignment
Patient 10
8000
6000
4000
2000
0
0
1000
2000
3000
4000
Fold Change over Starting Library
Amino Acid Number
A
B
Median
NS1
1000
800
600
400
200
0
700
800
900
1000
1100
1200
Fold Change over Starting Library
A
B
Me
Envelope
8000
6000
4000
2000
0
0
200
400
600
800
1000
Fold Change over Starting Library
Amino Acid Number
A
B
Median

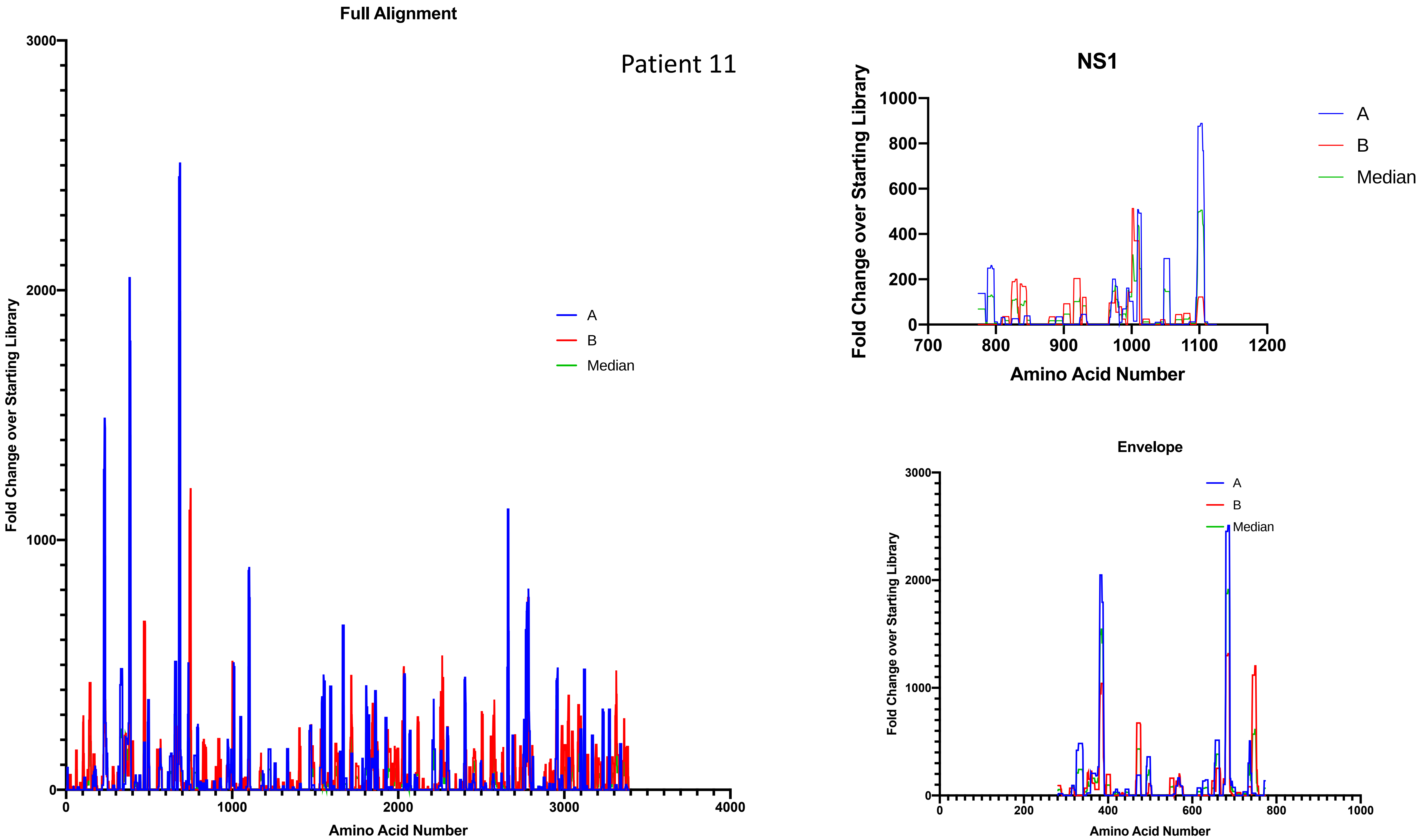
Full Alignment
Patient 11
Fold Change over Starting Library
Amino Acid Number
A
B
Median
0
1000
2000
3000
4000
NS1
Fold Change over Starting Library
Amino Acid Number
0
200
400
600
800
1000
700
900
1100
1200
Envelope
Fold Change over Starting Library
Amino Acid Number

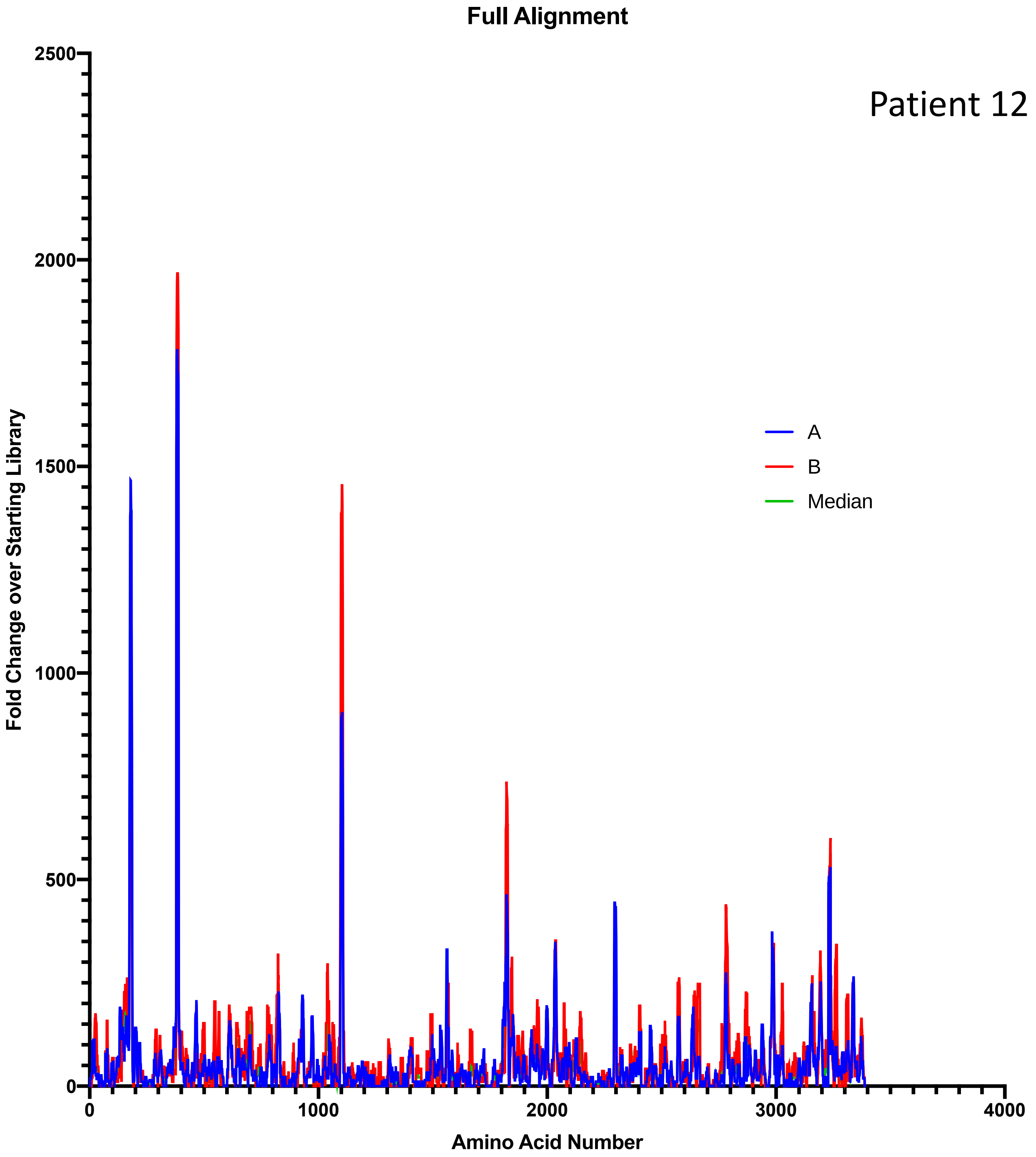

Full Alignment
Patient 12
Fold Change over Starting Library
Amino Acid Number
2500
2000
1500
1000
500
0
0
1000
2000
3000
4000
A
B
Median

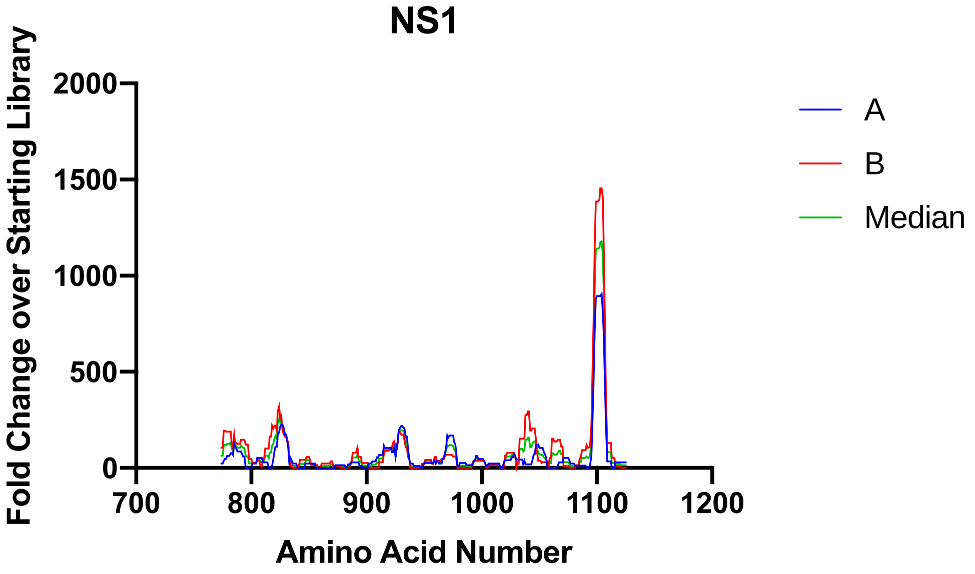

NS1
Fold Change over Starting Library
Amino Acid Number
2000
1500
1000
500
0
700
800
900
1000
1100
1200
A
B
Median

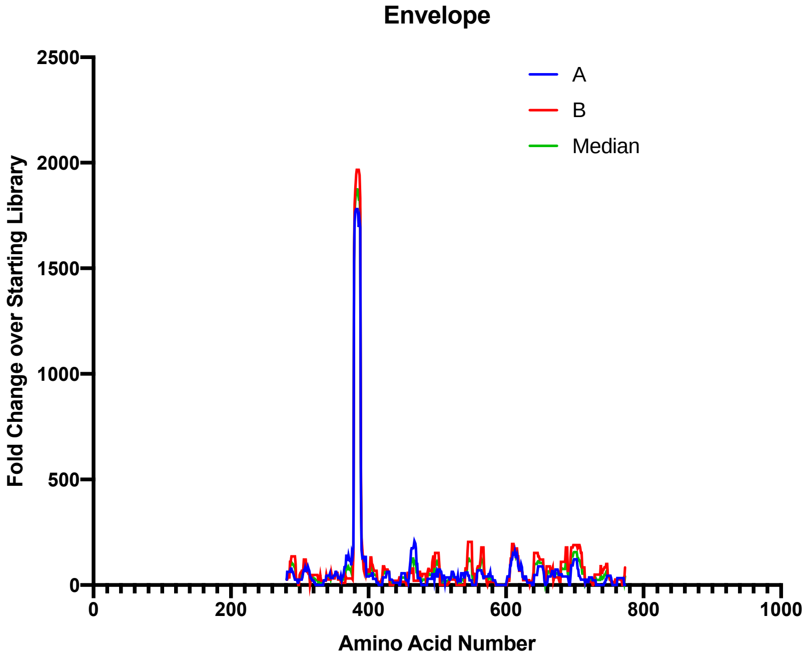

Envelope
Fold Change over Starting Library
Amino Acid Number
2500
2000
1500
1000
500
0
0
200
400
600
800
1000
A
B
Median

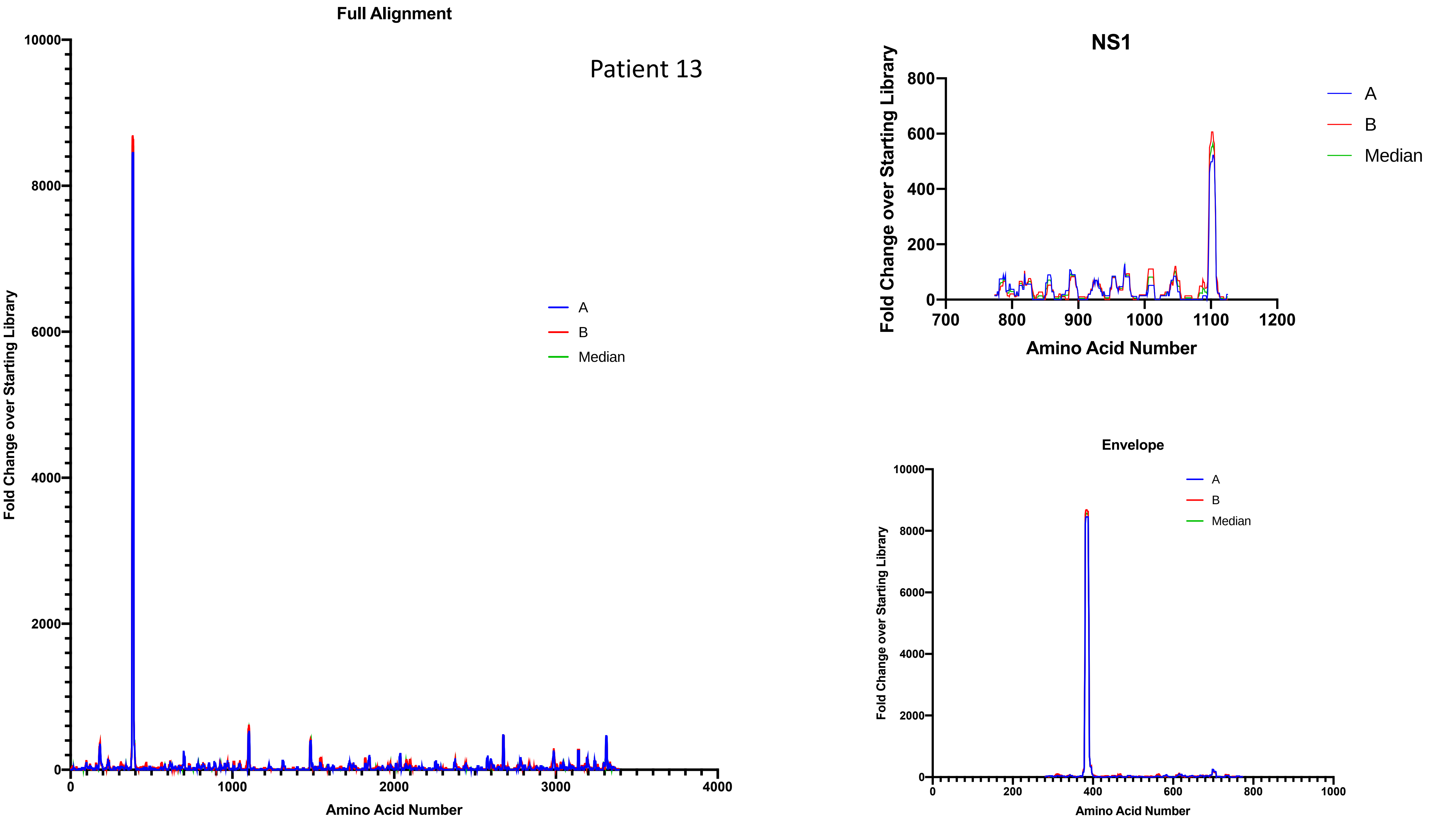

Full Alignment
Patient 13
Fold Change over Starting Library
Amino Acid Number
A
B
Median
0
2000
4000
6000
8000
10000
1000
3000
NS1
Fold Change over Starting Library
Amino Acid Number
200
400
600
800
700
800
900
1000
1100
1200
Envelope
Fold Change over Starting Library
Amino Acid Number
0
2000
4000
6000
8000
10000
200
400
600
800
1000

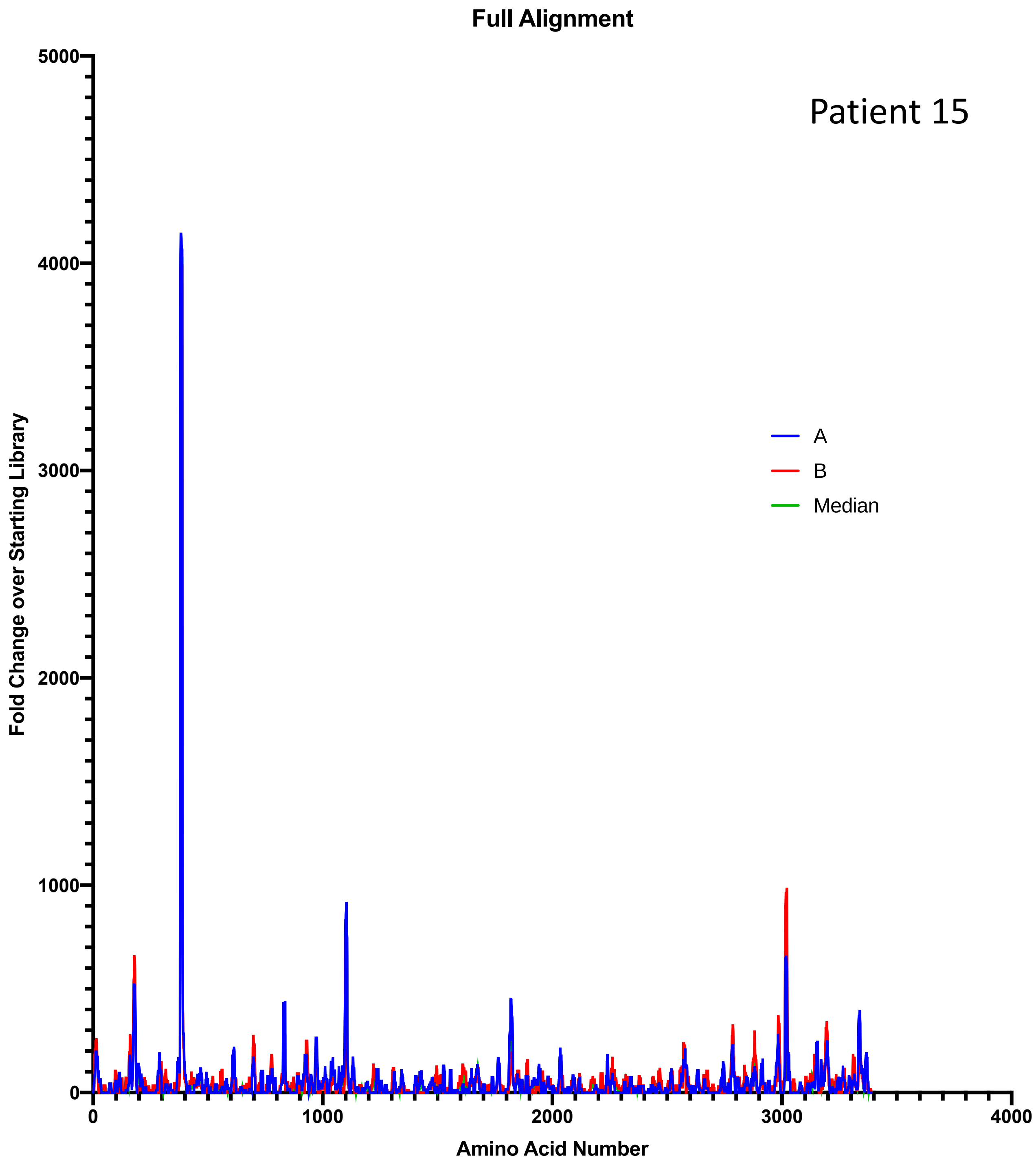
Full Alignment
Patient 15
Fold Change over Starting Library
Amino Acid Number
0
1000
2000
3000
4000
5000
A
B
Median

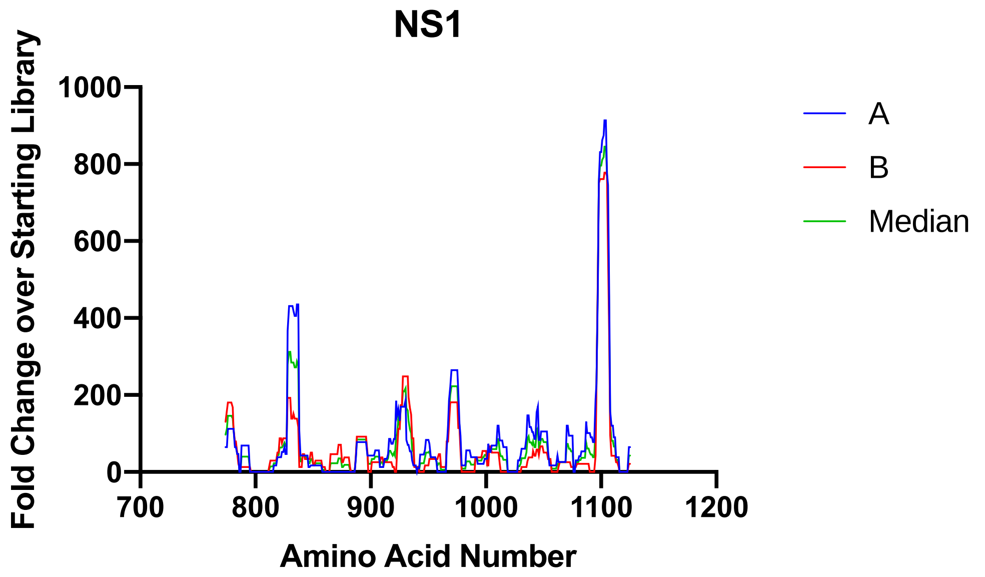
NS1
Fold Change over Starting Library
Amino Acid Number
0
200
400
600
800
1000
700
800
900
1000
1100
1200
A
B
Median

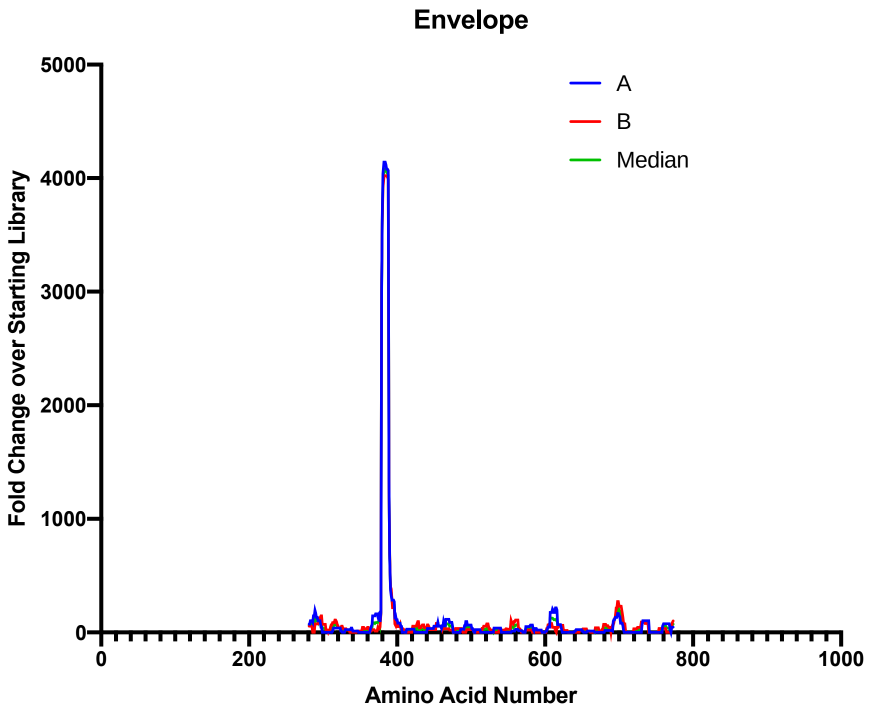
Envelope
Fold Change over Starting Library
Amino Acid Number
0
1000
2000
3000
4000
5000
200
400
600
800
A
B
Median

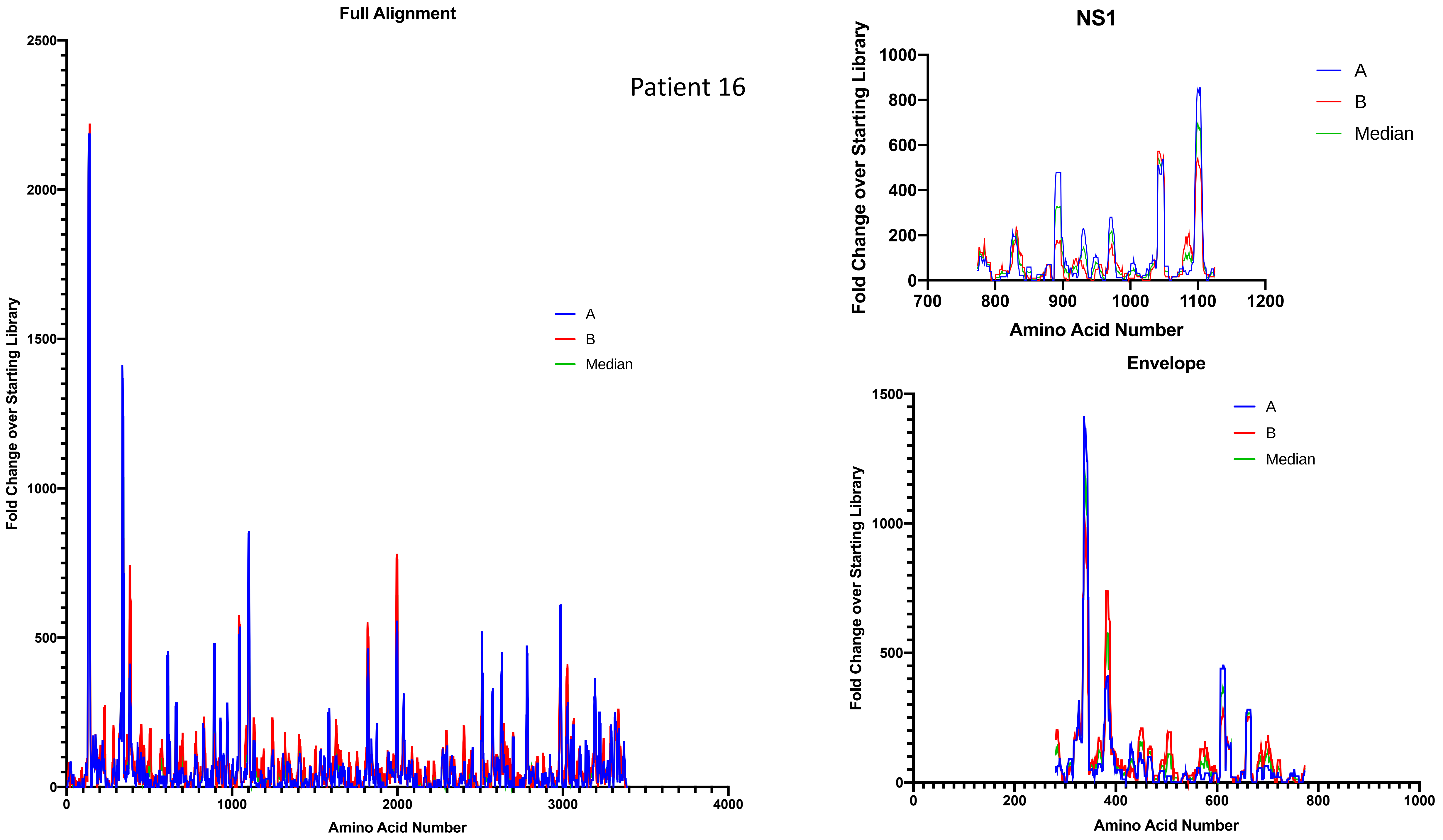
Full Alignment
Patient 16
Fold Change over Starting Library
2500
2000
1500
1000
500
0
0
1000
2000
3000
4000
Amino Acid Number
A
B
Median
NS1
Fold Change over Starting Library
1000
800
600
400
200
0
700
800
900
1000
1100
1200
Amino Acid Number
A
B
Median
Envelope
Fold Change over Starting Library
1500
1000
500
0
0
200
400
600
800
1000
Amino Acid Number
A
B
Median

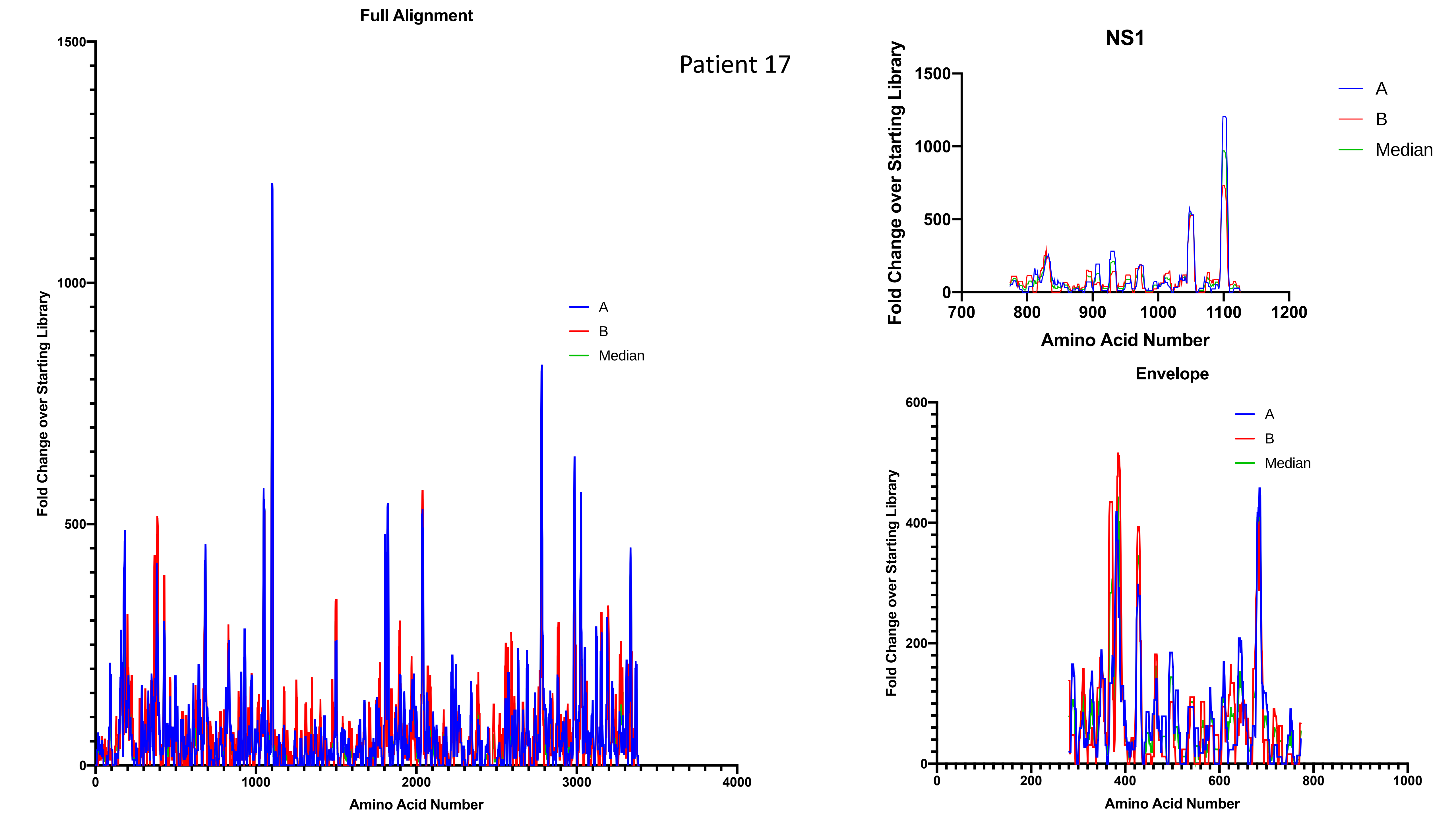
Full Alignment
Patient 17
Fold Change over Starting Library
Amino Acid Number
A
B
Median
NS1
Envelope

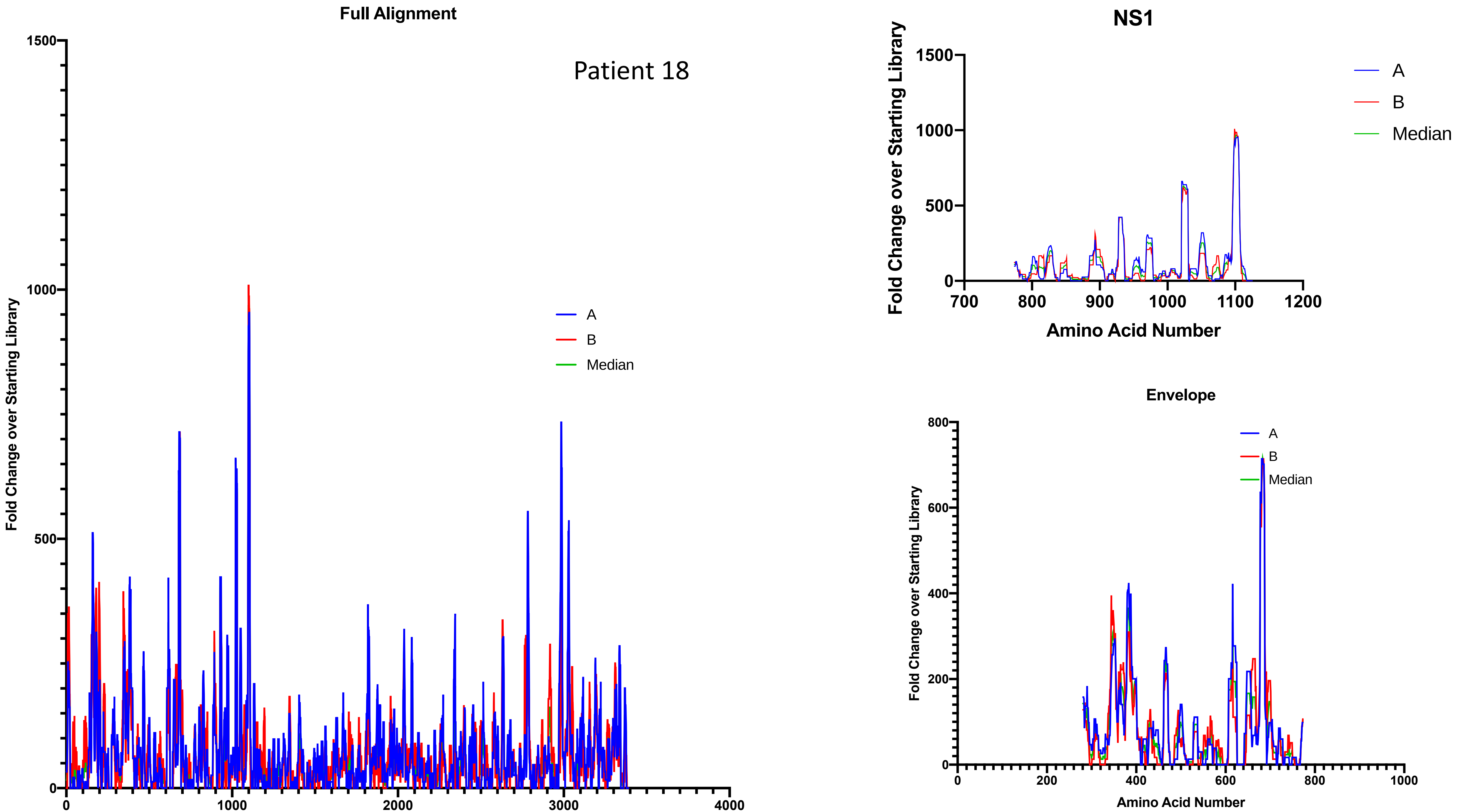
Full Alignment
Patient 18
Fold Change over Starting Library
1500
1000
500
0
0
1000
2000
3000
4000
A
B
Median
NS1
Fold Change over Starting Library
1500
1000
500
0
700
800
900
1000
1100
1200
Amino Acid Number
A
B
Median
Envelope
Fold Change over Starting Library
800
600
400
200
0
0
200
400
600
800
1000
Amino Acid Number
A
B
Median

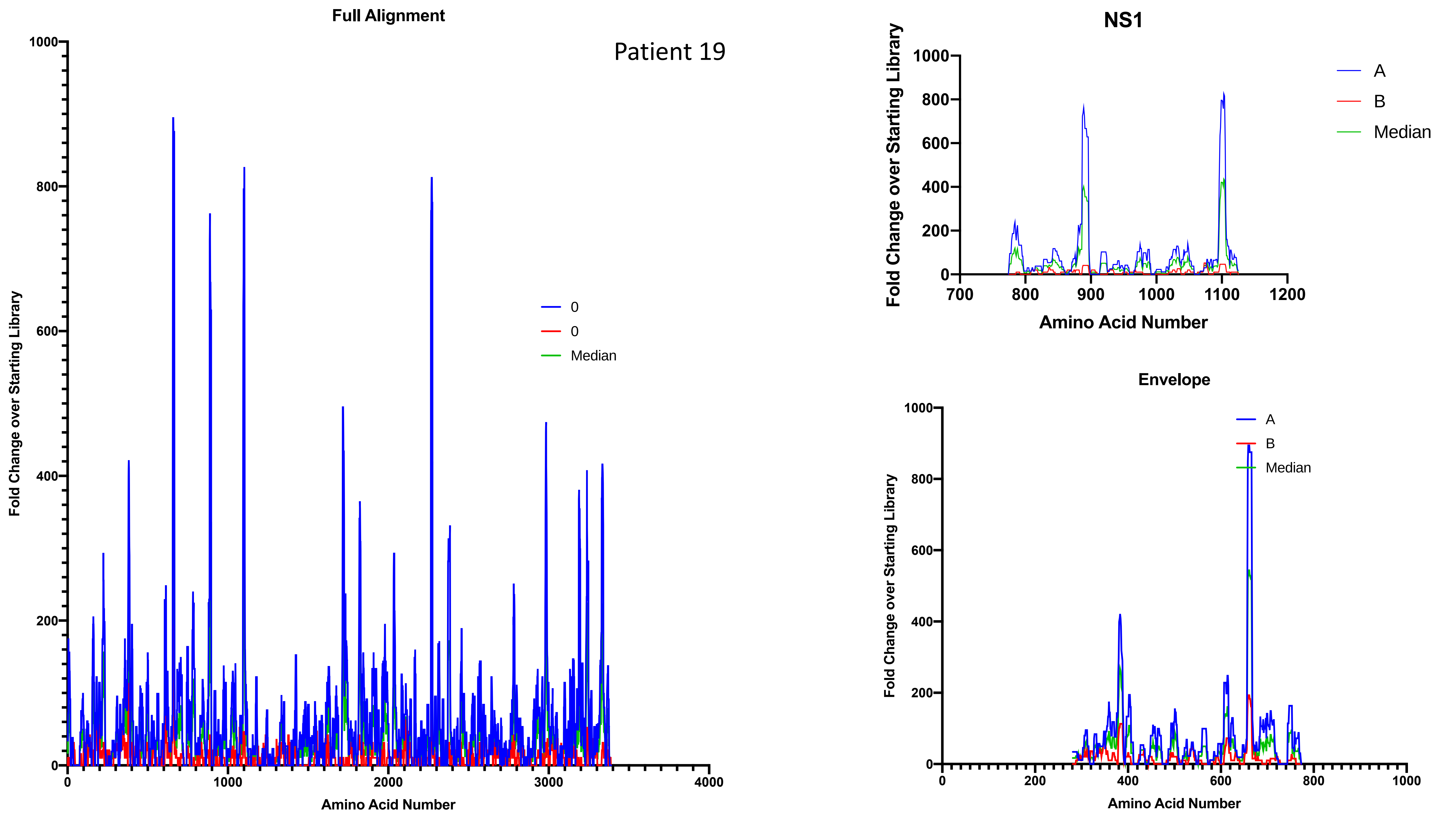

Full Alignment
Patient 19
0
0
Median
Fold Change over Starting Library
Amino Acid Number
NS1
A
B
Median
Fold Change over Starting Library
Amino Acid Number
Envelope
A
B
Median
Fold Change over Starting Library
Amino Acid Number

Patient 20

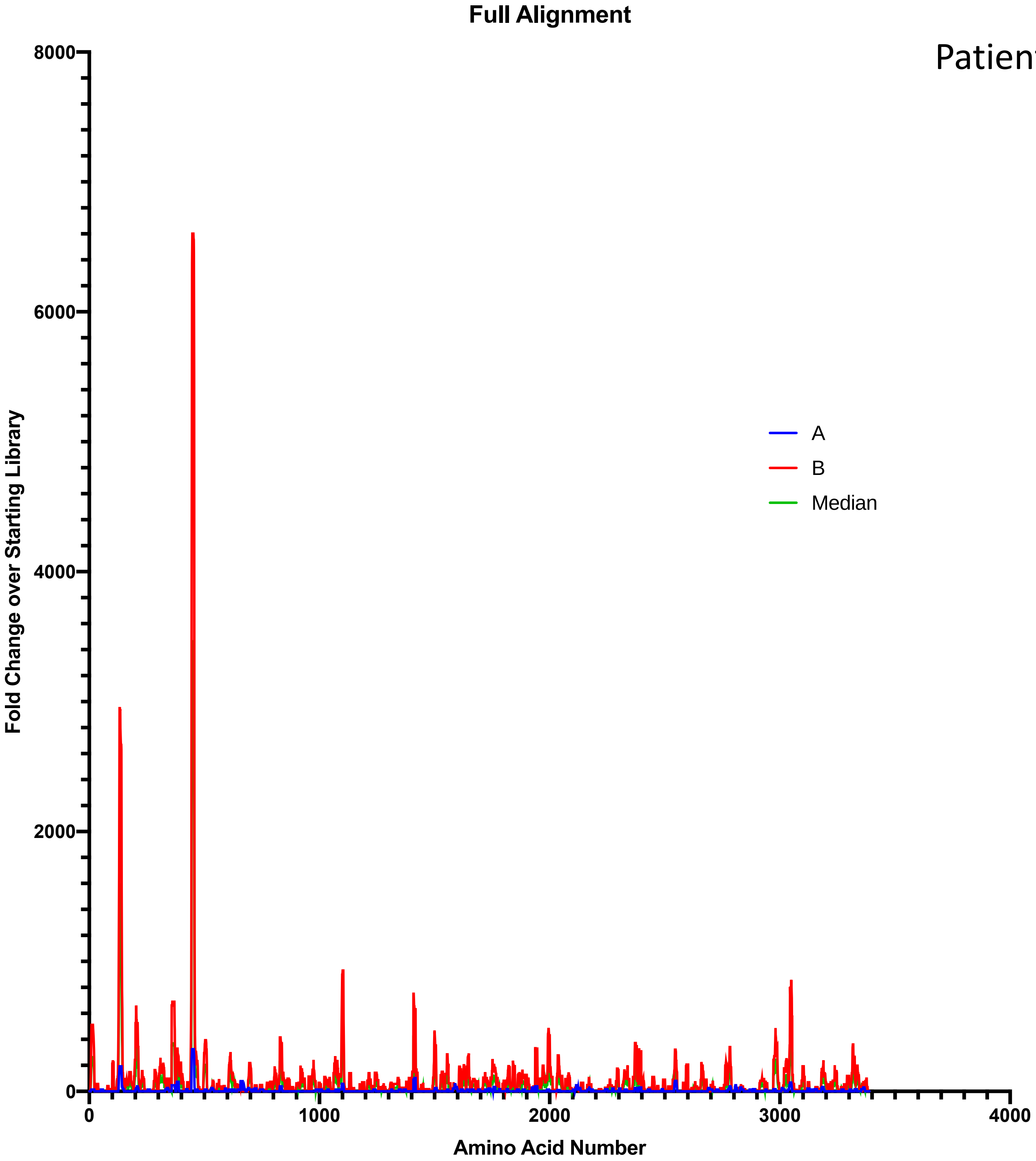

Full Alignment
Fold Change over Starting Library
Amino Acid Number
0
2000
4000
6000
8000
0
1000
2000
3000
4000
A
B
Median

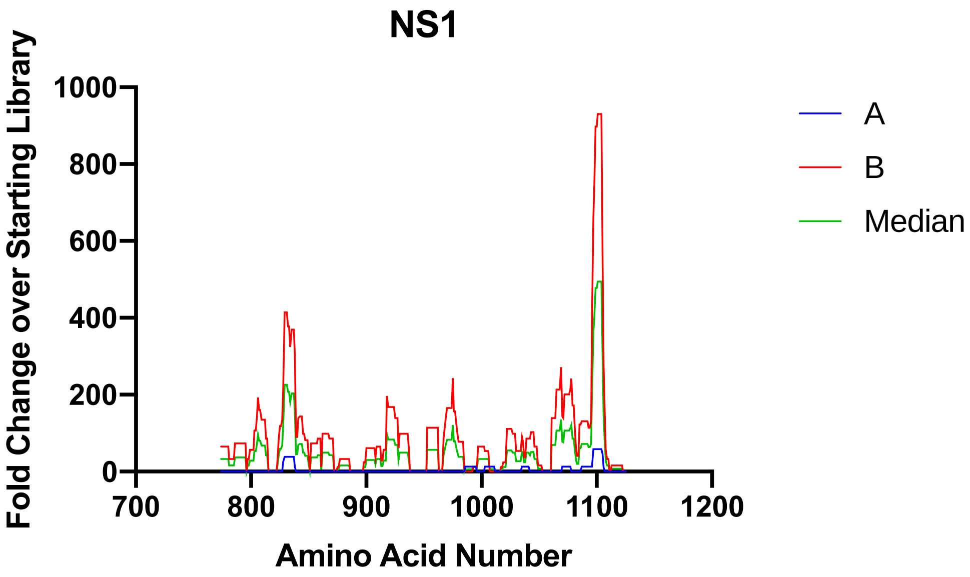

NS1
Fold Change over Starting Library
Amino Acid Number
0
200
400
600
800
1000
700
800
900
1000
1100
1200
A
B
Median

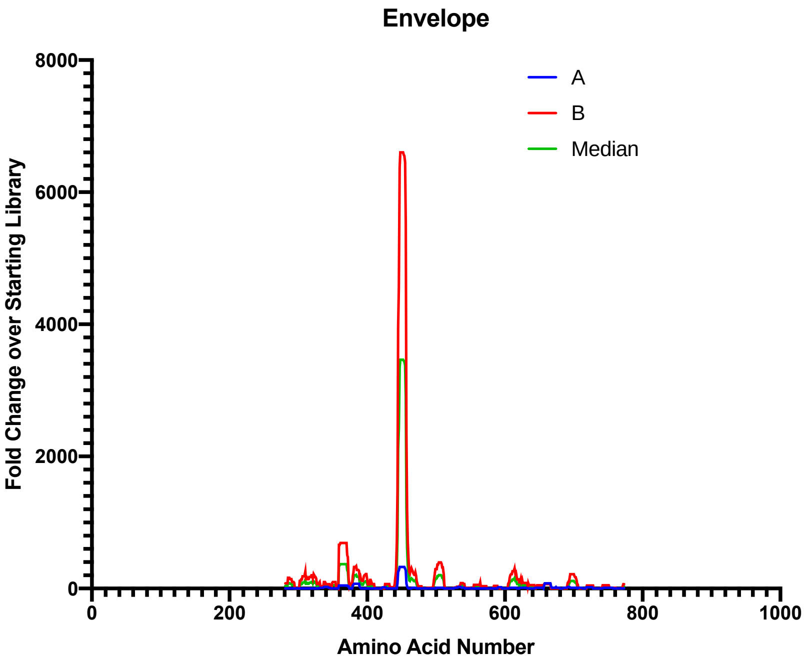

Envelope
Fold Change over Starting Library
Amino Acid Number
0
2000
4000
6000
8000
0
200
400
600
800
1000
A
B
Median

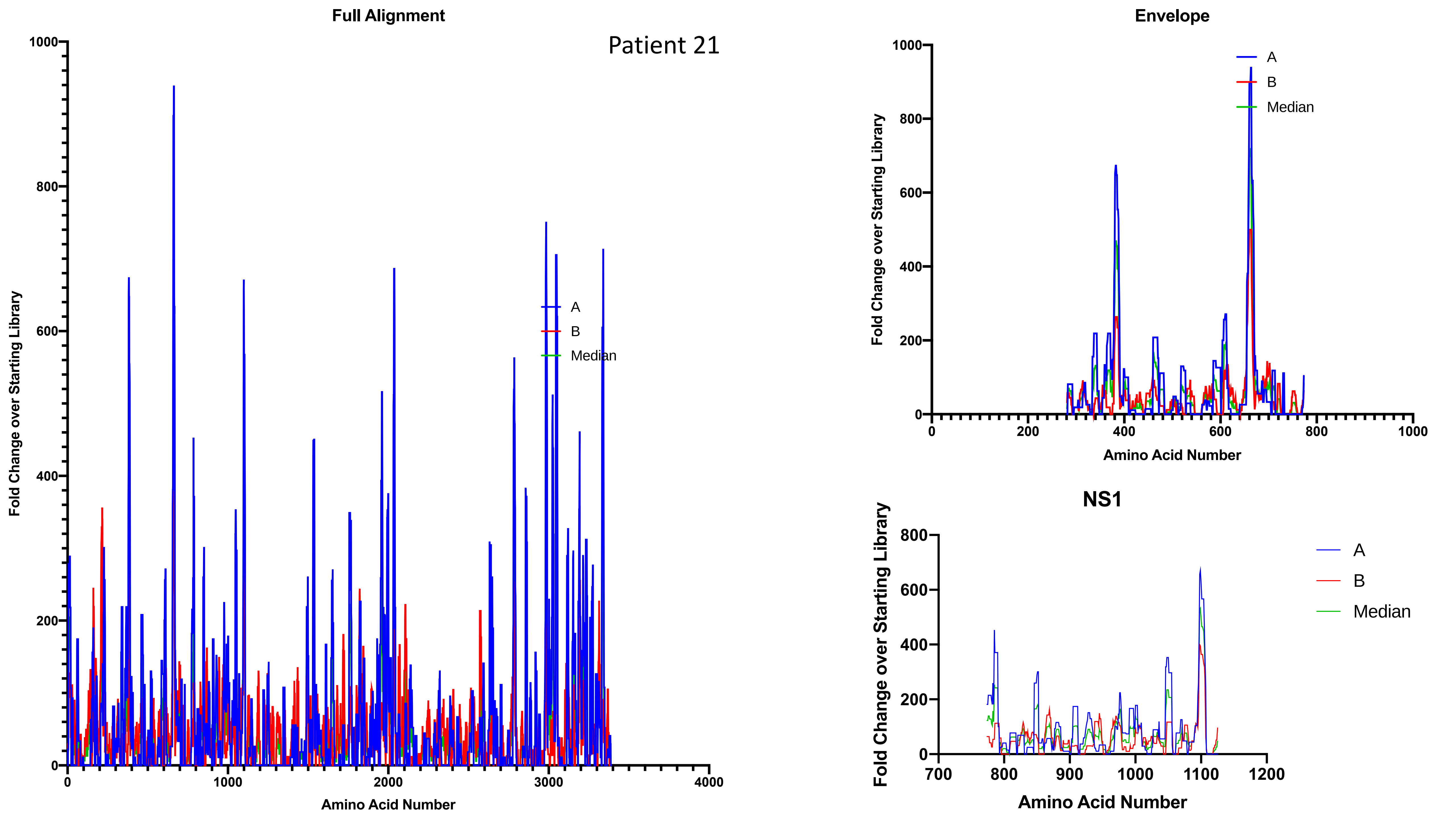
Full Alignment
Patient 21
Fold Change over Starting Library
Amino Acid Number
A
B
Median
0
200
400
600
800
1000
0
1000
2000
3000
4000
Envelope
Fold Change over Starting Library
Amino Acid Number
A
B
Median
0
200
400
600
800
1000
NS1
Fold Change over Starting Library
Amino Acid Number
A
B
Median
0
200
400
600
800
700
800
900
1000
1100
1200

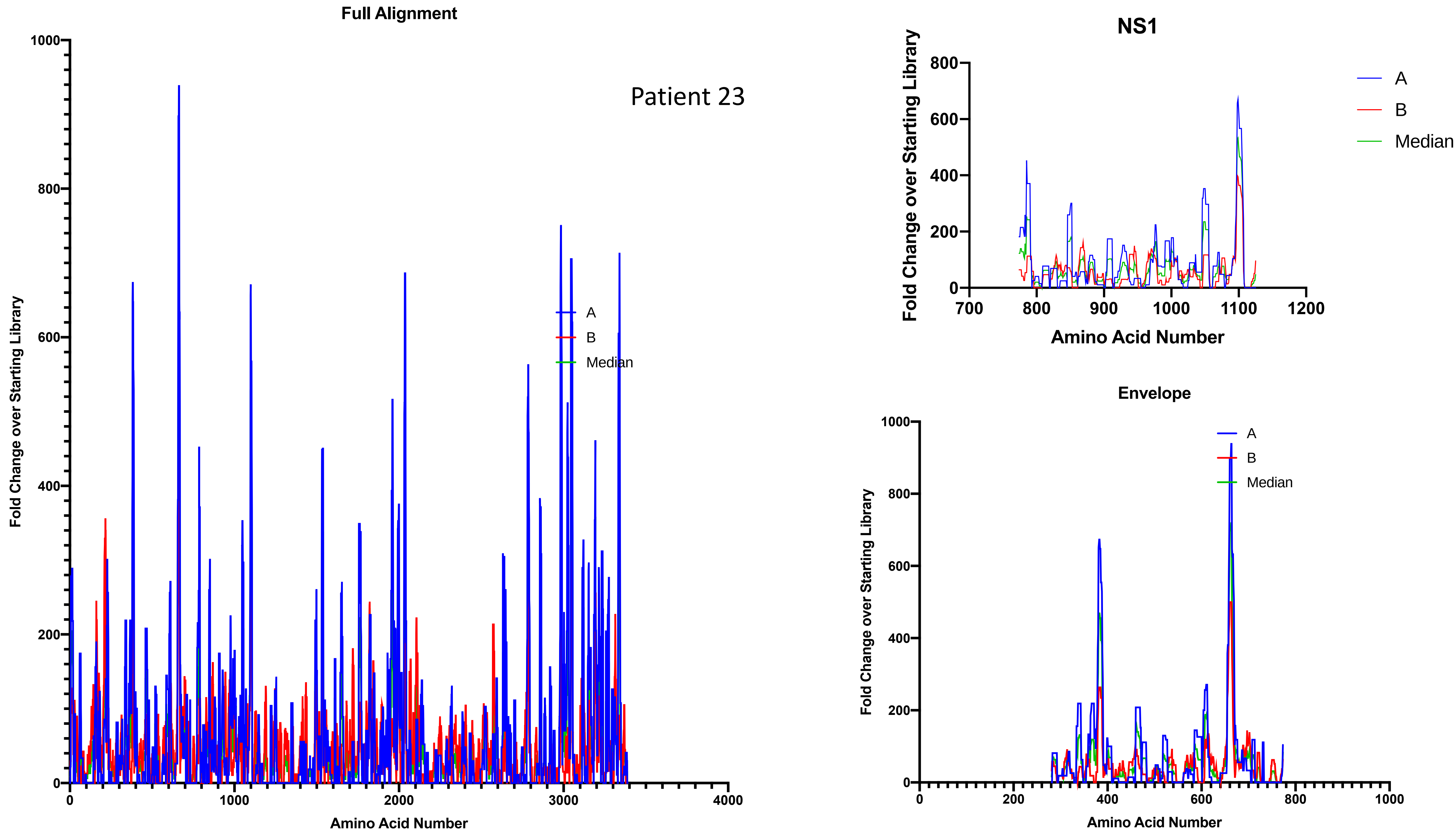
Full Alignment
Patient 23
Fold Change over Starting Library
Amino Acid Number
A
B
Median
NS1
Fold Change over Starting Library
Amino Acid Number
A
B
Median
Envelope
Fold Change over Starting Library
Amino Acid Number
A
B
Median

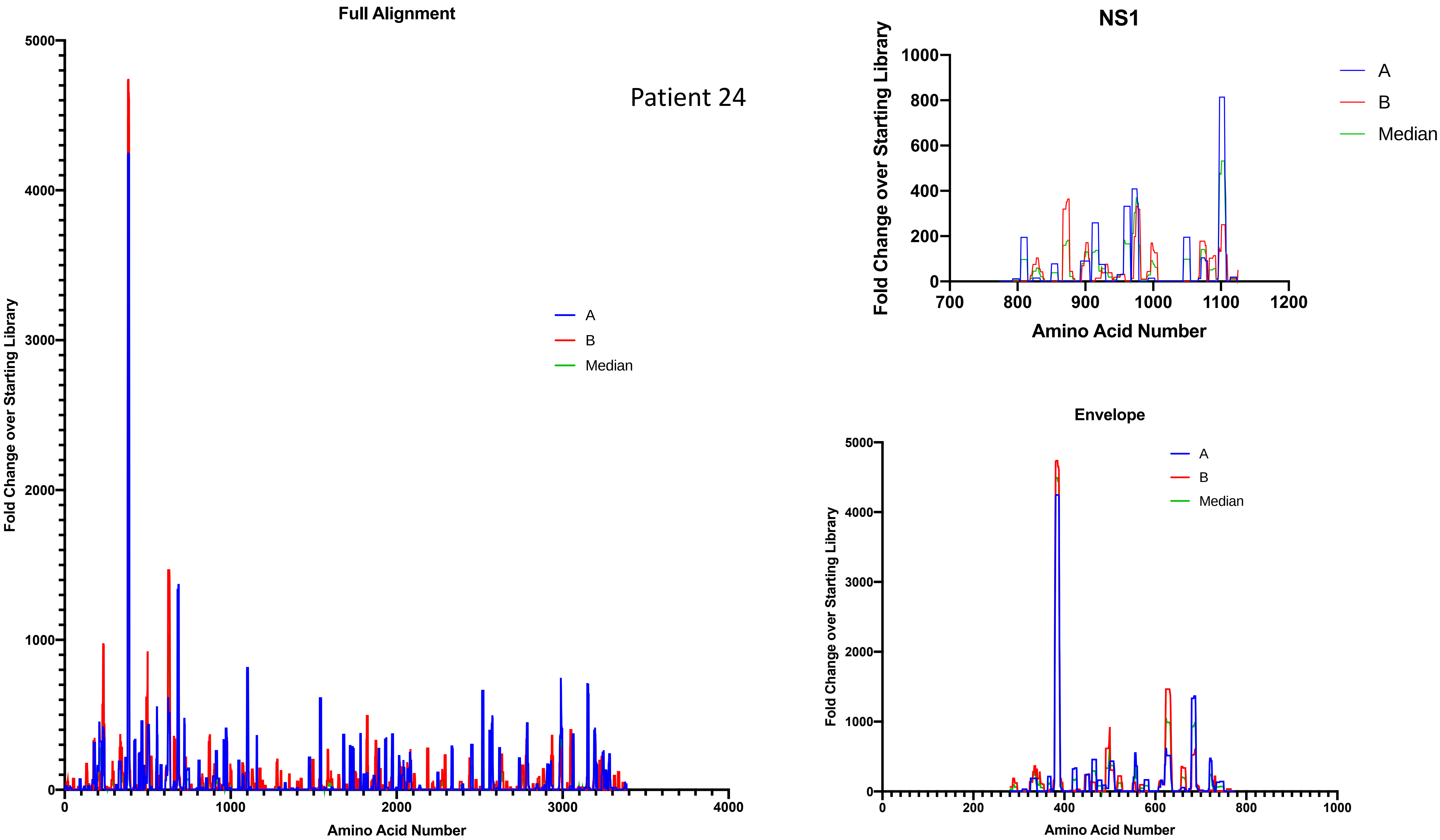

Full Alignment
Patient 24
Fold Change over Starting Library
Amino Acid Number
A
B
Median
0
1000
2000
3000
4000
5000
NS1
Fold Change over Starting Library
Amino Acid Number
700
800
900
1000
1100
1200
0
200
400
600
800
1000
A
B
Median
Envelope
Fold Change over Starting Library
Amino Acid Number
0
200
400
600
800
1000
2000
3000
4000
5000
A
B
Median

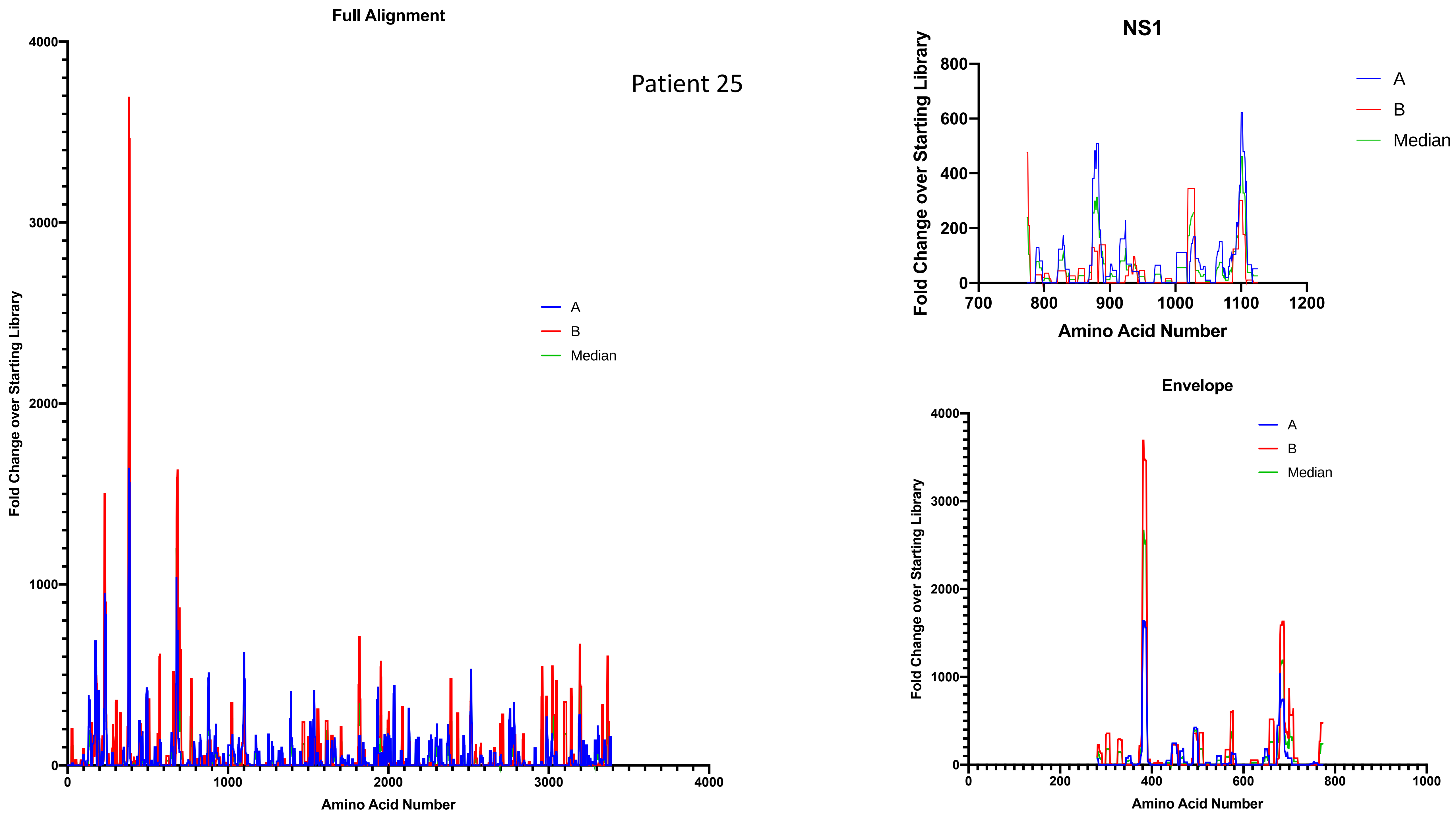

Full Alignment
Patient 25
Fold Change over Starting Library
Amino Acid Number
A
B
Median
0
1000
2000
3000
4000
NS1
Fold Change over Starting Library
Amino Acid Number
0
200
400
600
800
700
800
900
1000
1100
1200
A
B
Median
Envelope
Fold Change over Starting Library
Amino Acid Number
0
1000
2000
3000
4000
0
200
400
600
800
1000
A
B
Median

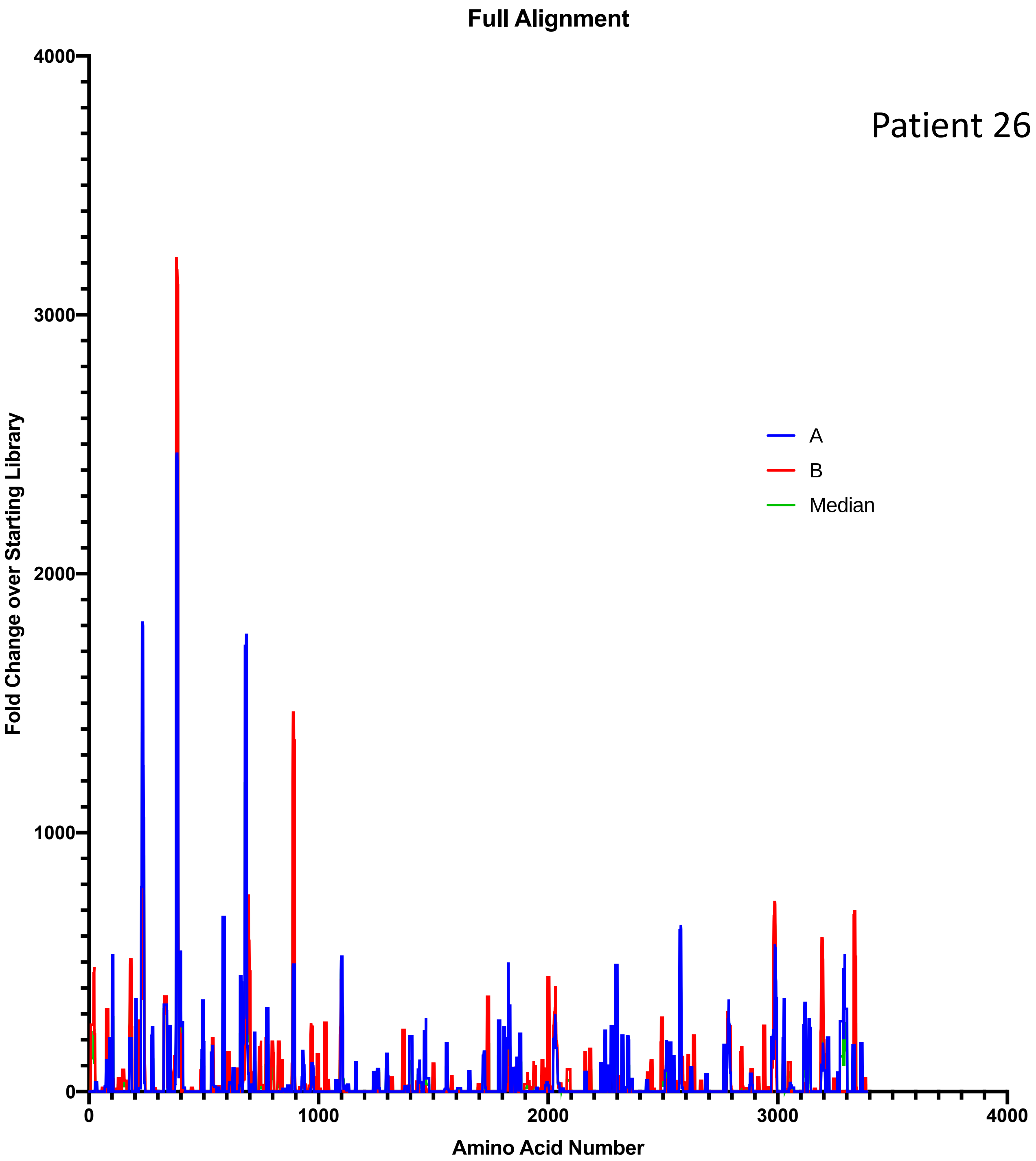
Full Alignment
Patient 26
A
B
Median
Fold Change over Starting Library
Amino Acid Number
0
1000
2000
3000
4000

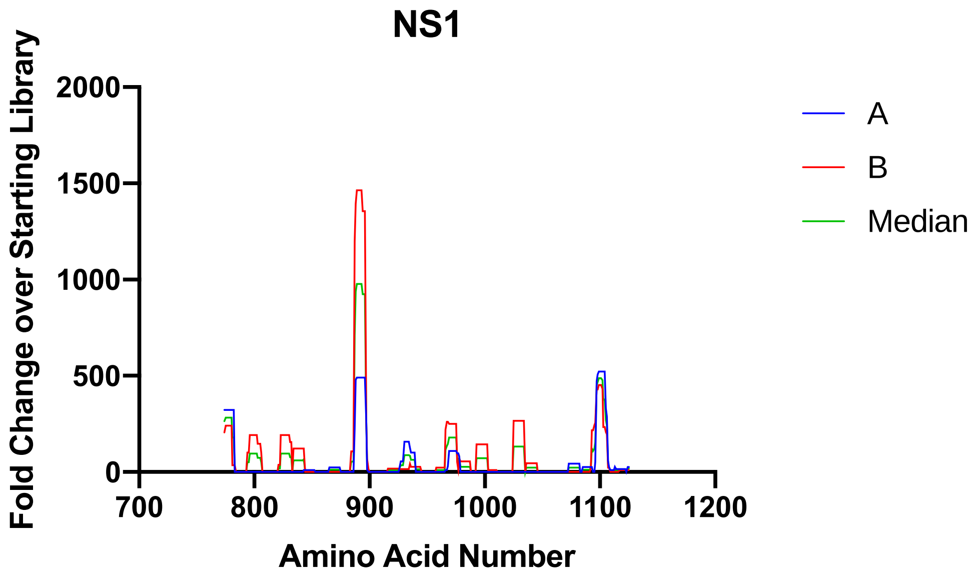
NS1
A
B
Median
Fold Change over Starting Library
Amino Acid Number
0
500
1000
1500
2000
700
800
900
1000
1100
1200

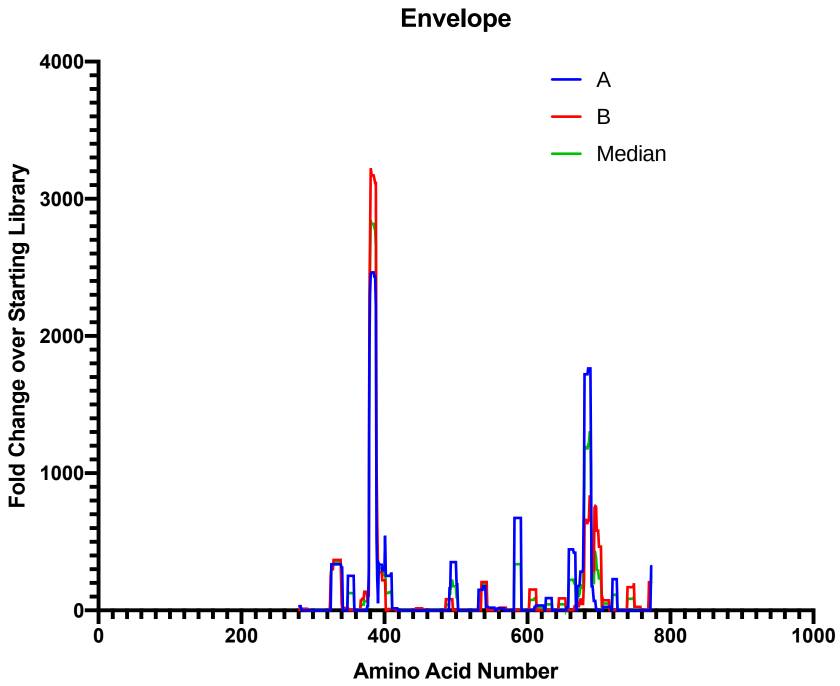
Envelope
A
B
Median
Fold Change over Starting Library
Amino Acid Number
0
1000
2000
3000
4000
200
400
600
800

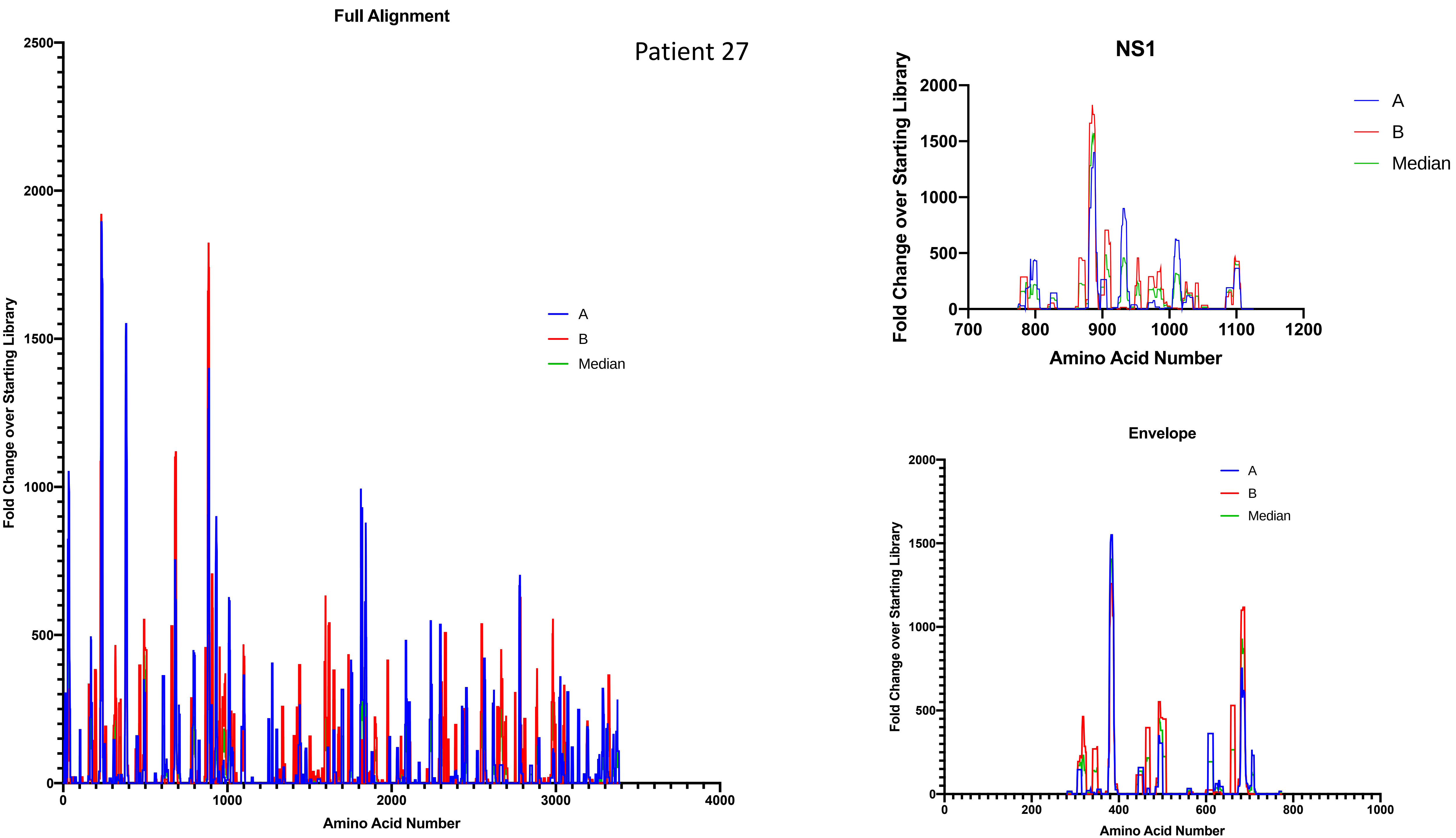
Full Alignment
Patient 27
Fold Change over Starting Library
Amino Acid Number
A
B
Median
0
500
1000
1500
2000
2500
0
1000
2000
3000
4000
NS1
Fold Change over Starting Library
Amino Acid Number
0
500
1000
1500
2000
700
800
900
1000
1100
1200
A
B
Median
Envelope
Fold Change over Starting Library
Amino Acid Number
0
500
1000
1500
2000
0
200
400
600
800
1000
A
B
Median

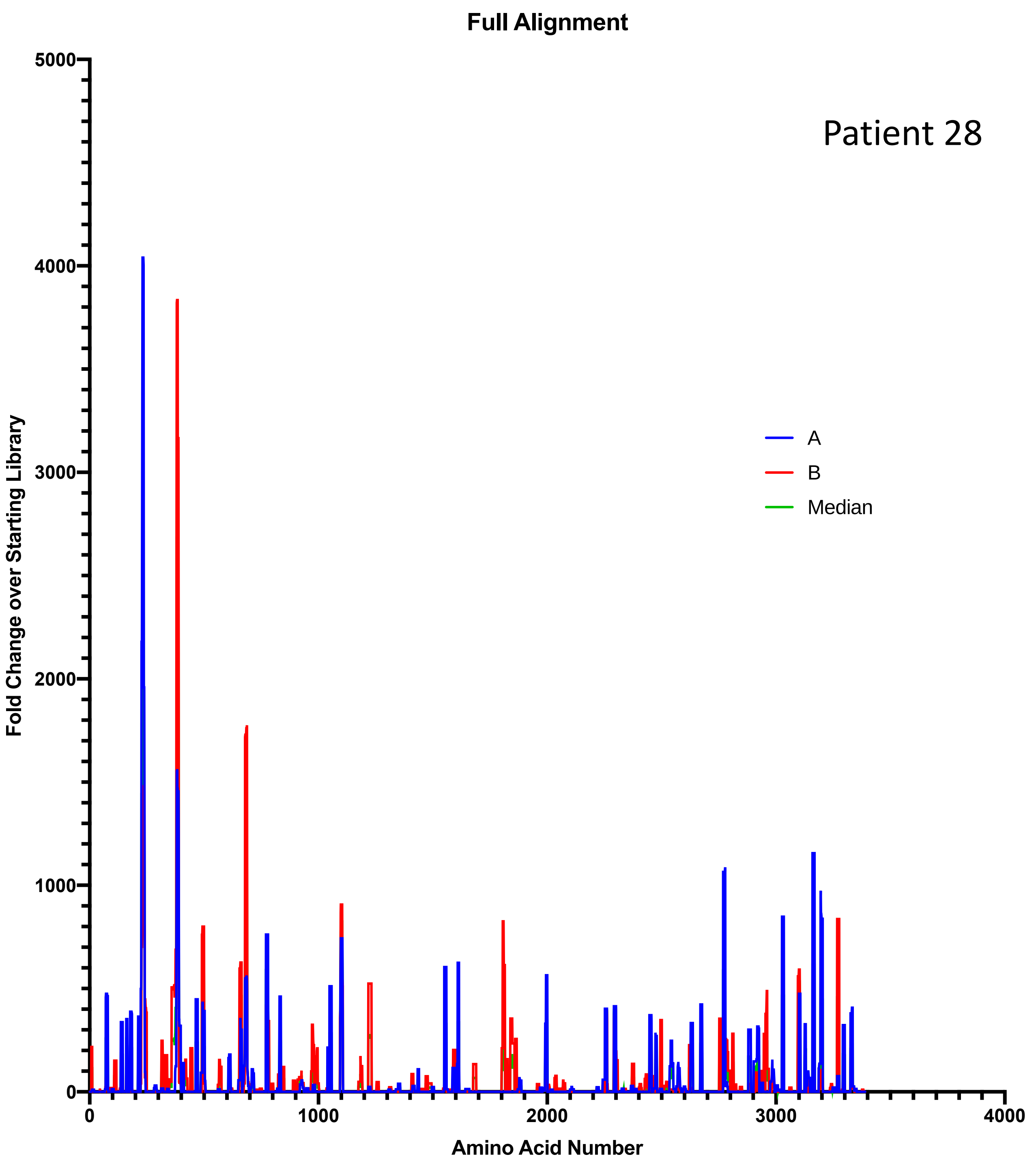

Full Alignment
Patient 28
5000
4000
3000
2000
1000
0
Fold Change over Starting Library
A
B
Median
0
1000
2000
3000
4000
Amino Acid Number

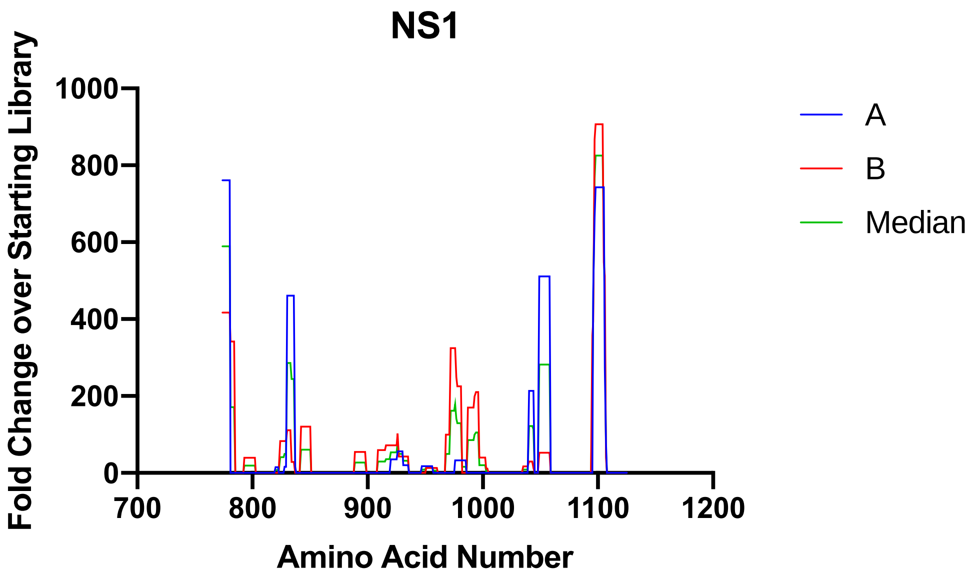

NS1
1000
800
600
400
200
0
Fold Change over Starting Library
700
800
900
1000
1100
1200
Amino Acid Number
A
B
Median

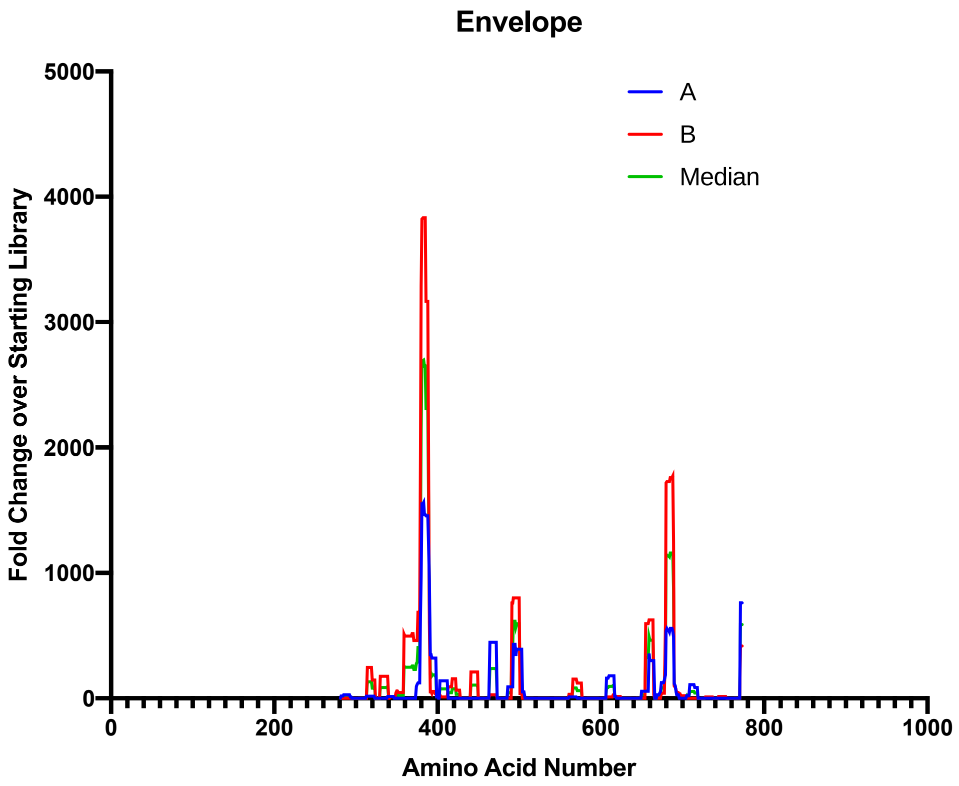

Envelope
5000
4000
3000
2000
1000
0
Fold Change over Starting Library
A
B
Median
0
200
400
600
800
1000
Amino Acid Number
